# A genetic screen implicates a CWC16/Yju2/CCDC130 protein and SMU1 in alternative splicing in *Arabidopsis thaliana*

**DOI:** 10.1261/rna.060517.116

**Published:** 2017-07

**Authors:** Tatsuo Kanno, Wen-Dar Lin, Jason L. Fu, Antonius J.M. Matzke, Marjori Matzke

**Affiliations:** Institute of Plant and Microbial Biology, Academia Sinica, Taipei 115, Taiwan

**Keywords:** alternative splicing, CWC16, SmF, SMU1, Yju2

## Abstract

To identify regulators of pre-mRNA splicing in plants, we developed a forward genetic screen based on an alternatively spliced *GFP* reporter gene in *Arabidopsis thaliana*. In wild-type plants, three major splice variants issue from the *GFP* gene but only one represents a translatable *GFP* mRNA. Compared to wild-type seedlings, which exhibit an intermediate level of *GFP* expression, mutants identified in the screen feature either a “GFP-weak” or “Hyper-GFP” phenotype depending on the ratio of the three splice variants. GFP-weak mutants, including previously identified *prp8* and *rtf2*, contain a higher proportion of unspliced transcript or canonically spliced transcript, neither of which is translatable into GFP protein. In contrast, the coilin-deficient *hyper-gfp1* (*hgf1*) mutant displays a higher proportion of translatable *GFP* mRNA, which arises from enhanced splicing of a U2-type intron with noncanonical AT–AC splice sites. Here we report three new *hgf* mutants that are defective, respectively, in spliceosome-associated proteins SMU1, SmF, and CWC16, an Yju2/CCDC130-related protein that has not yet been described in plants. The *smu1* and *cwc16* mutants have substantially increased levels of translatable *GFP* transcript owing to preferential splicing of the U2-type AT–AC intron, suggesting that SMU1 and CWC16 influence splice site selection in *GFP* pre-mRNA. Genome-wide analyses of splicing in *smu1* and *cwc16* mutants revealed a number of introns that were variably spliced from endogenous pre-mRNAs. These results indicate that SMU1 and CWC16, which are predicted to act directly prior to and during the first catalytic step of splicing, respectively, function more generally to modulate splicing patterns in plants.

## INTRODUCTION

Splicing of precursor mRNA (pre-mRNA) through excision of noncoding regions (introns) and joining of adjacent coding regions (exons) is essential for the expression of nearly all eukaryotic protein-coding genes. Splicing is catalyzed by the spliceosome, a large and dynamic ribonucleoprotein (RNP) machine located in the nucleus. Spliceosomes comprise five small nuclear (sn) RNPs, each containing a heptameric ring of Sm or Sm-like proteins and a different snRNA (U1, U2, U4, U5, or U6), as well as numerous other non-snRNP proteins. During the splicesomal reaction cycle, the five snRNPs act sequentially on the pre-mRNA with a changing assemblage of non-snRNP proteins to form a series of complexes that catalyze two consecutive transesterification reactions (Wahl et al. 2009; Will and Lührmann 2011; Matera and Wang 2014; Meyer 2016). The U1 and U2 snRNPs first recognize the 5′ and 3′ splice sites and conserved branch points of introns and interact to form pre-spliceosomal complex A. The subsequent addition of preformed U4/U5/U6 tri-snRNP creates pre-catalytic complex B. Ensuing reorganization steps induce release of U1 and U4 snRNPs and conversion of complex B to complex B*, which catalyzes the first reaction yielding the free 5′ exon and lariat 3′-exon intermediates. Newly formed C complex catalyzes the second reaction to achieve intron lariat excision and exon ligation (Wahl et al. 2009; Will and Lührmann 2011; Matera and Wang 2014; Meyer 2016). Lastly, dismantling of the spliceosome frees individual components to assemble anew at the next intron.

The spliceosome is responsible for both constitutive and alternative splicing (Matera and Wang 2014). Constitutive splicing occurs when the same splice sites are always used, resulting in a single mature transcript from a given gene. In contrast, alternative splicing involves variable usage of splice sites and selective removal of introns from multi-intron pre-mRNAs (Reddy et al. 2012). Alternative splicing leads to the production of multiple mature RNAs from a single primary transcript, thereby increasing transcriptome and proteome diversity (Matera and Wang 2014). Major modes of alternative splicing include intron retention, exon skipping, and alternative 5′ and/or 3′ splice site choice (Marquez et al. 2012). In animals, the main outcome of alternative splicing is exon skipping, whereas in plants, intron retention predominates (Kornblihtt et al. 2013). Only 5% of genes in budding yeast contain introns and alternative splicing is rare (Naftelberg et al. 2015; Gould et al. 2016). In contrast, most animal and plant genes comprise multiple introns and the majority undergoes alternative splicing (Nilsen and Graveley 2010; Marquez et al. 2012). Alternative splicing has roles in regulating gene expression during development of multicellular organisms (Staiger and Brown 2013; Zhang et al. 2016) and is important for stress adaptation in plants (Ali and Reddy 2008; Filichkin et al. 2015).

The biochemical mechanisms that regulate alternative splicing are complex and only partially understood (Nilsen and Graveley 2010; Reddy et al. 2013). Selection of alternative splice sites is influenced by exonic and intronic *cis*-regulatory elements known as splicing enhancers and silencers, which bind *trans*-acting splicing factors such as SR (serine/arginine-rich) proteins and hnRNPs (heterogeneous ribonucleoproteins) (Barta et al. 2008; Matera and Wang 2014; Meyer 2016; Sveen et al. 2016). Certain tissue-specific factors and core spliceosomal proteins are able to regulate alternative splicing (Nilsen and Graveley 2010; Saltzman et al. 2011). Changes in chromatin structure can modulate patterns of alternative splicing by affecting transcription rates and hence splice site selection (Naftelberg et al. 2015). However, much remains to be learned about the full array of factors responsible for determining alternative splicing patterns in higher organisms (Kornblihtt et al. 2013; Reddy et al. 2013).

The protein composition and structure of the major U2-type spliceosome at various stages of the splicing process have been studied primarily in budding yeast and metazoans. The spliceosome of budding yeast comprises 50–60 core snRNP subunits and around 100 additional splicing-associated proteins, most of which are conserved in higher eukaryotes (Koncz et al. 2012). Reflecting the more complex splicing requirements of multicellular eukaryotes, spliceosomes in *Drosophila melanogaster* and humans contain several hundred largely conserved proteins (Fabrizio et al. 2009; Herold et al. 2009; Agafonov et al. 2011; Will and Lührmann 2011). Although less is known about splicesome composition in plants, the *Arabidopsis* genome is predicted to encode approximately 430 conserved spliceosomal factors, indicating a degree of structural and mechanistic complexity comparable to that observed in metazoans (Koncz et al. 2012). Notably, around half of the conserved homologs are duplicated in *Arabidopsis*, potentially allowing functional diversification and evolution of plant-specific functions (Koncz et al. 2012; Reddy et al. 2012).

Although inventories of core spliceosomal proteins and auxiliary splicing components have been compiled for *Arabidopsis* (Koncz et al. 2012; Reddy et al. 2013), their mechanistic roles in splicing often remain unclear. Lack of an in vitro splicing system in plants has impeded functional analyses of predicted splicing proteins (Reddy et al. 2012). To identify proteins that impact splicing efficiency and alternative splicing in plants, we are performing a forward genetic screen based on an alternatively spliced *GFP* reporter gene in *Arabidopsis*. The usefulness of this genetic system has been validated by the identification of a novel factor, RTF2 (Replication Termination Factor2), which may participate in ubiquitin-based regulation of the spliceosome (Sasaki et al. 2015), and the finding of an unexpected role for the Cajal body marker protein coilin in attenuating splicing efficiency of a small subset of stress-related genes (Kanno et al. 2016). Here we report three new mutants identified in the screen that are defective, respectively, in splicing factors SMU1, SmF, and CWC16, which are related to budding yeast first step factor Yju2 and human CCDC130. We present evidence indicating that SMU1 and CWC16, which have not yet been studied in plants, can influence splice site selection and alternative splicing patterns in *Arabidopsis*.

## RESULTS

### Forward genetic screen and identification of new hgf mutants

**FIGURE 1.**
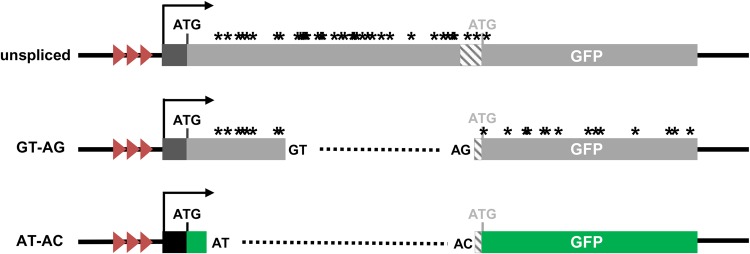
Alternatively spliced *GFP* reporter gene. The *GFP* reporter gene, which is under the transcriptional control of viral regulatory elements, features a GT–AG intron nested inside a U2-type intron with noncanonical AT–AC splice sites. Three major splice variants accumulate from the *GFP* reporter gene: a long unspliced transcript, a middle-length transcript resulting from splicing of the GT–AG intron, and a shorter transcript resulting from splicing of the AT–AC intron. Only the latter is translatable into GFP protein (indicated by green colored bar). The short black bar represents the promoter region. The transcription start site is indicated by the black arrow. Red arrowheads indicate a tandem repeat in the enhancer region. Asterisks denote premature termination codons. The black ATG indicates the main translation initiation codon. The downstream gray stippled area and ATG represent an unused promoter and initiation codon, respectively. The 3′ splice sites for the GT–AG and AT–AC introns are separated by only 3 nt with the noncanonical AC on the *outside* (Sasaki et al. 2015; Kanno et al. 2016).

The forward genetic screen to identify factors influencing pre-mRNA splicing in plants exploits a transgenic *Arabidopsis* “T” line containing an alternatively spliced *GFP* reporter gene (referred to hereafter as “wild type”). Of three major *GFP* splice variants observed in wild-type plants, only one, which results from splicing a U2-type intron with noncanonical AT–AC splice sites, gives rise to a translatable *GFP* mRNA. The other two transcripts—a spliced *GFP* transcript resulting from removal of a canonical GT–AG intron and an unspliced *GFP* pre-mRNA—are not translatable owing to the presence of many premature termination codons after the initiating methionine (Fig. 1; Kanno et al. 2016). Our working hypothesis is that mutations in genes encoding splicing factors will change the ratio of the three transcripts and hence either increase or decrease *GFP* mRNA levels. Such changes will result, respectively, in either a “Hyper-GFP” or “GFP-weak” phenotype relative to the wild-type T line, which has an intermediate level of GFP fluorescence (Kanno et al. 2016).

Following ethyl methanesulfonate (EMS) mutagenesis of seeds of the wild-type T line, we screened M2 seedlings for mutants displaying either enhanced or diminished GFP fluorescence. In addition to around a dozen GFP-weak (*gfw*) complementation groups, we recovered approximately 10 Hyper-GFP (*hgf*) complementation groups including *hgf1*, which comprises mutants defective in the Cajal body marker protein coilin (At1g13030) (Kanno et al. 2016).

**FIGURE 2.**
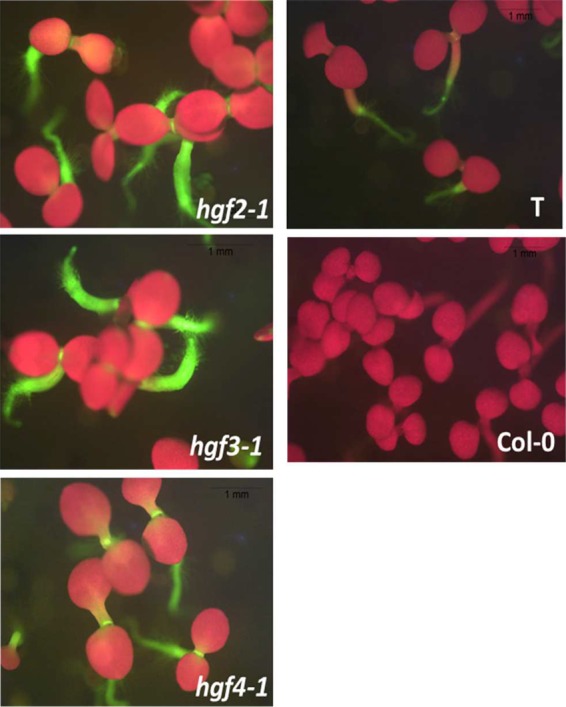
Hyper-GFP phenotype of new *hgf* mutants. Appearance of ∼2-wk-old seedlings of *hgf2-1*, *hgf3-1*, and *hgf4-1* mutants as well as the wild-type T line and untransformed Col-0 growing on solid MS medium as visualized under a fluorescence stereo microscope. In the *hgf* mutants, GFP fluorescence is considerably increased in the seedling stem and shoot apex, which is visible between the two seedling leaves (these appear red owing to auto-fluorescence of chlorophyll at the excitation wavelength of GFP).

**FIGURE 3.**
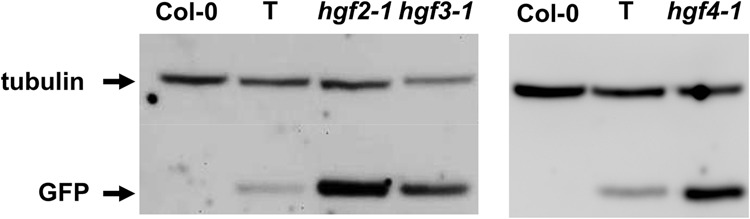
Western blot analysis of GFP protein in *hgf* mutants. Total proteins were extracted from ∼2-wk-old seedlings, separated by SDS-PAGE, and blotted onto a PVDF membrane. The blot was probed sequentially with antibodies to GFP protein and tubulin as a loading control (Fu et al. 2015). Sample names are indicated at the *top*. Separate lanes for the wild-type T line and nontransgenic Col-0 are shown for *hgf* mutant samples that were run on separate gels.

**FIGURE 4.**
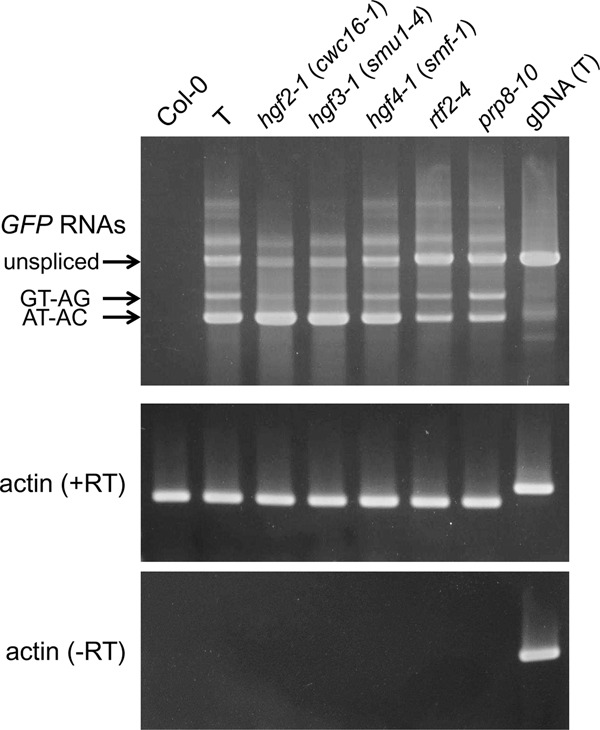
RT-PCR of *GFP* splice variants. Semi-quantitative RT-PCR was used to examine the accumulation of the unspliced *GFP* transcript and the two spliced transcripts (resulting from splicing the canonical GT–AG and noncanonical AT–AC introns, respectively) in the indicated *hgf* and *gfw* mutants, the wild-type T line, and nontransgenic Col-0. The *gfw*1 and *gfw2* mutants represent new alleles of *rtf2* and *prp8*, respectively (Supplemental Fig. S1). Actin is shown as a constitutively expressed control. –RT, no reverse transcriptase. gDNA (T), genomic DNA isolated from T line.

Here we report three new *hgf* mutants: *hgf2-1*, *hgf3-1*, and *hgf4-1*, which all display a characteristic Hyper-GFP phenotype in seedlings (Fig. 2). Western blotting using an antibody to GFP confirmed that the enhanced fluorescence is due to increased amounts of GFP protein (Fig. 3). Semi-quantitative RT-PCR was used to estimate the levels of the three *GFP* splice variants in the mutants relative to wild type. In *hgf2-1* and *hgf3-1* mutants, levels of translatable *GFP* transcript were elevated and levels of untranslatable canonically spliced and unspliced transcripts were reduced (Fig. 4). In the *hgf4-1* mutant, the levels of the three splice variants remained approximately at wild-type levels. In contrast, in two *gfw* mutants, which harbor new alleles of previously identified *prp8* and *rtf2* (Supplemental Fig. S1), the level of the translatable transcript was reduced while the level of the unspliced, untranslatable transcript was increased (Fig. 4).

To identify the causal mutations in *hgf2-1*, *hgf3-1*, and *hgf4-1*, we performed next-generation mapping (NGM) using DNA isolated from pools of F2 progeny exhibiting a Hyper-GFP phenotype (James et al. 2013). Analysis of NGM data revealed that the three new *hgf* mutants have mutations in genes encoding known splicing factors that are either uncharacterized or not yet studied extensively in *Arabidopsis*: *hgf2* corresponds to At1g25682, which encodes coiled-coil domain-containing protein Yju2/CWC16/CCDC130; *hgf3* corresponds to At1g73720, which encodes WD40 repeat-containing protein SMU1 (suppressor of mec-8 and unc-52 1); and *hgf4* corresponds to At4g30220, which encodes the snRNP protein SmF (small nuclear ribonucleoprotein F). Complementation tests, in which each *hgf* mutant was transformed with a construct containing the respective wild-type cDNA sequence under the control of the endogenous transcriptional regulatory sequences, confirmed the identity of the mutated genes (Supplemental Fig. S2). The three homozygous *hgf* mutants were viable and fertile under standard growth conditions.

### hgf2: Yju2/CWC16/CCDC130

**FIGURE 5.**
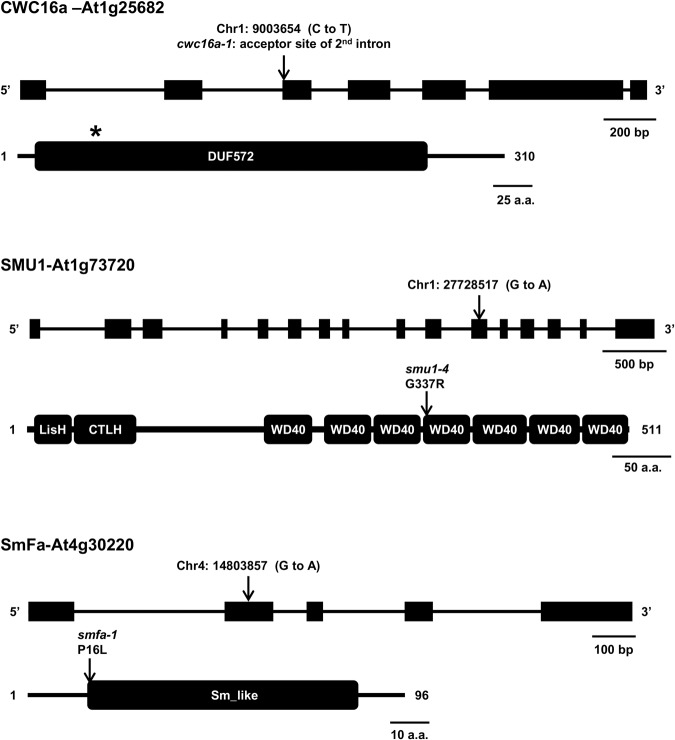
Positions of mutations in *hgf* mutants and protein domain structure. The mutated nucleotide is shown *above* the intron–exon structure of each gene. The resulting amino acid substitution or premature termination codon (PTC) is indicated *above* the protein domain structure. (*Top*) CWC16a is a 310 amino acid protein containing a DUF527 (domain of unknown function). The *cwc16a-1* allele destroys the acceptor site of the second intron, disrupting the open reading frame, and creating a PTC (asterisk) around 50 amino acids into the protein (Supplemental Fig. S5). (*Middle*) SMU1 is a 511 amino acid protein containing seven WD40 repeats as well as N-terminal LisH (lis homology) and CTLH (C-terminal to LisH domain) domains, which may promote dimerization. The *smu1-4* mutation results in a G337R substitution in one of the WD40 repeats. (*Bottom*) SmFa is a 96 amino acid protein comprising an Sm-like domain. The *smfa-1* mutation results in a P16L substitution at the beginning of the Sm-like domain.

The CWC16 gene family, which encodes proteins consisting largely of a domain of unknown function (DUF527), is evolutionarily conserved in plants (Supplemental Fig. S3) and other eukaryotes (Supplemental Fig. S4). The mutation we identified is likely a null, destroying the acceptor site of the second intron and disrupting the open reading frame after approximately 50 amino acids (Fig. 5; Supplemental Fig. S5).

In *Arabidopsis*, CWC16 proteins are encoded by a previously uncharacterized gene family that contains six members, including two paralogs that are full-length and expressed: At1g25682 (identified in our screen) and At1g17130, which will be referred to hereafter as CWC16a and CWC16b, respectively. In addition, there are three unexpressed retroposed genes (retrogenes) derived from CWC16b (Zhang et al. 2005), and one truncated, unexpressed gene that is most similar to CWC16a (Table 1; Supplemental Fig. S5). The two expressed *CWC16* genes correspond to two classes of CWC16 family members, which are represented in humans by coiled-coil domain-containing proteins CCDC130 and CCDC94. CWC16a and its truncated paralog are orthologous to the CCDC130 class whereas CWC16b and the three related retrogenes are most similar to CCDC94 and to budding yeast splicing factor Yju2 (Table 1). Apart from budding yeast and a few fungi and protozoa, which have only CCDC94 orthologs, most eukaryotes have orthologs of both CCDC94 and CCDC130 (Supplemental Table S1). The mutant allele we identified, which is the first reported for At1g25628, is designated *cwc16a-1* (Fig. 5). Whether CWC16b can functionally compensate to any extent for the presumed null *cwc16a-1* mutation is not known.

**TABLE 1.**
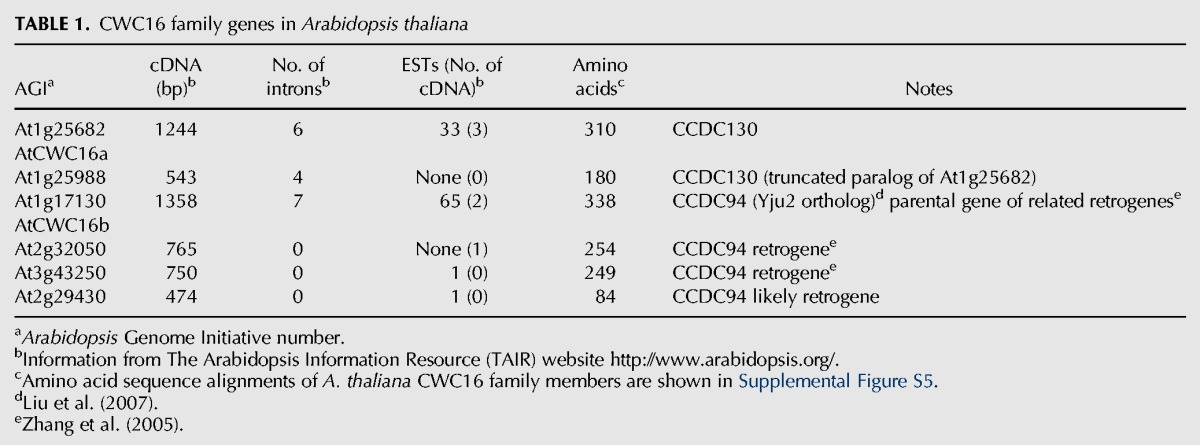
CWC16 family genes in *Arabidopsis thaliana*

### hgf3: SMU1

In *Arabidopsis*, SMU1 is encoded by a single copy gene, At1g73720. The mutation we identified creates a Gly337Arg substitution in the WD40 domain (Fig. 5). It is unknown whether this is a complete loss-of-function mutation although a similar substitution of a glycine residue in the WD40 domain of Smu1 in hamster cells causes a temperature-sensitive loss of function (Sugaya et al. 2005). In view of three previously reported T-DNA insertion mutants of *SMU1* in *Arabidopsis* (*smu1-1*, *smu1-2*, and *smu1-3*) (Chung et al. 2009), we designate our allele *smu1-4* (Fig. 5).

### hgf4: SMF

SmF is a core protein of spliceosomal snRNPs. The mutation we identified creates a P16L substitution (Fig. 5), which affects a conserved proline residue important for forming the heptamer interface with six other Sm proteins in the snRNP ring (https://www.ncbi.nlm.nih.gov/). SmF is encoded by duplicated genes in *Arabidopsis*: At4g30220 (identified in our screen) and At2g14285 (Cao et al. 2011), which are referred to hereafter as *SMFa* and *SMFb*, respectively. *SMFa* is also termed *RUXF* (http://www.arabidopsis.org/). The mutant allele we identified, which is the first reported for At4g30220, is named *smfa-1* (Fig. 5).

### Genome-wide analysis of alternative splicing

**FIGURE 6.**
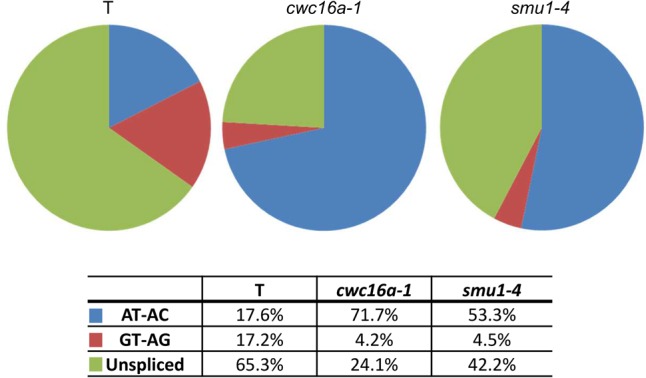
Abundance of *GFP* splice variants in the *cwc16a-1* and *smu1-4* mutants. Percentages of the three major splice variants of *GFP* RNA were determined from an analysis of RNA-seq data (Supplemental Table S11, data from biological replicate [3]). Total *GFP* transcript levels did not change significantly in the mutants (Supplemental Table S2).

We used RNA sequencing (RNA-seq) to study the genome-wide effects of the *cwc16a-1* and *smu1-4* mutations on splicing patterns and differential gene transcription. These two mutants were chosen for detailed analysis because they had the most obvious effect on splicing of the *GFP* reporter gene as determined by semi-quantitative RT-PCR (Fig. 4). Analysis of the RNA-seq data confirmed the RT-PCR data by demonstrating preferential splicing of the AT–AC intron in the *GFP* pre-mRNA to produce the translatable *GFP* transcript. Although the total *GFP* transcript level did not change significantly in the *cwc16a-1* and *smu1-4* mutants (Supplemental Table S2), the proportion of translatable transcripts resulting from splicing the AT–AC intron increased to over 50% in both mutants compared to only around 17.5% for the wild-type T line (Fig. 6; Supplemental Table S3). Conversely, the amount of canonically spliced, untranslatable transcript in the mutants decreased to around a quarter of the wild-type level. Splicing of the AT–AC intron was thus enhanced whereas splicing of the GT–AG intron was reduced in the mutants. The level of unspliced transcript in the mutants also decreased relative to the wild-type level, particularly in *cwc16a-1* (Fig. 6; Supplemental Table S3).

**FIGURE 7.**
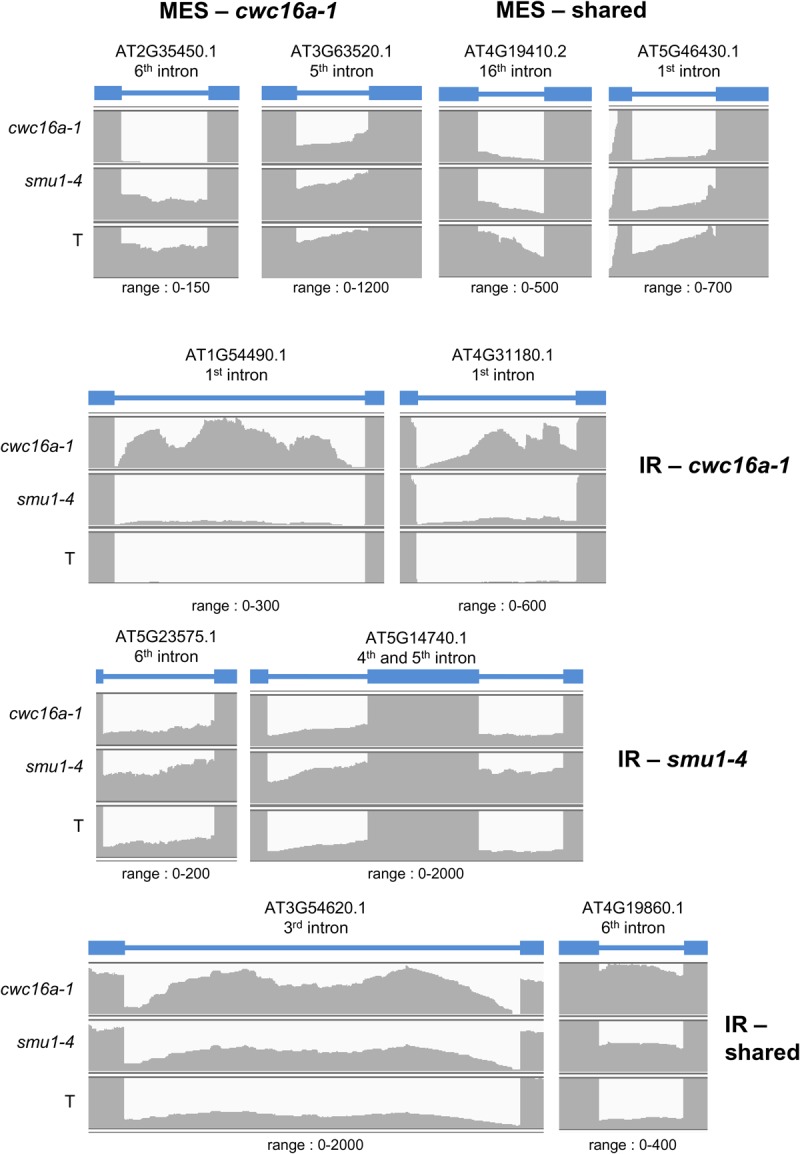
Examples of introns affected in splicing efficiency in *cwc16a-1* and *smu1-4* mutants. Read numbers of representative introns showing either more efficient splicing (MES; *top*) or increased intron retention (IR; *middle* and *bottom*) in *cwc16a-1* and *smu1-4* mutants or both (shared) compared to the wild-type T line are visualized by the Integrative Genomic Viewer (http://software.broadinstitute.org/software/igv/). The target intron and the two flanking exons are indicated by the blue bars and blue boxes, respectively. The *Arabidopsis* Genome Initiative (AGI) number for the target intron-containing gene and the range for counting the reads are shown *above* and *below* each display. The genes shown here are not among those identified as differentially expressed genes in the mutant lines.

Splicing of endogenous pre-mRNAs was affected in the mutants although the number of genes affected was not exceptionally high (Table 2). In the *smu1-4* mutant, intron retention (IR) was the most common occurrence. The 91 IR events in *smu1-4* included 13 cases (comprising 34 IR events in total) in which more than one intron was retained in a single pre-mRNA. In contrast, there were no cases of multiple MES events affecting a single gene in *smu1-4* (Table 2A; Supplemental Table S4). In the *cwc16a-1* mutant, the numbers of IR and MES events were roughly similar (Table 2A), and no instances of multiple introns being affected in a single gene were observed in either category (Supplemental Table S4). The number of shared IR and MES events in the two mutants was low (Table 2A; Supplemental Table S4). Examples of IR and MES events in each mutant compared to wild type are shown schematically in Figure 7.

**TABLE 2.**
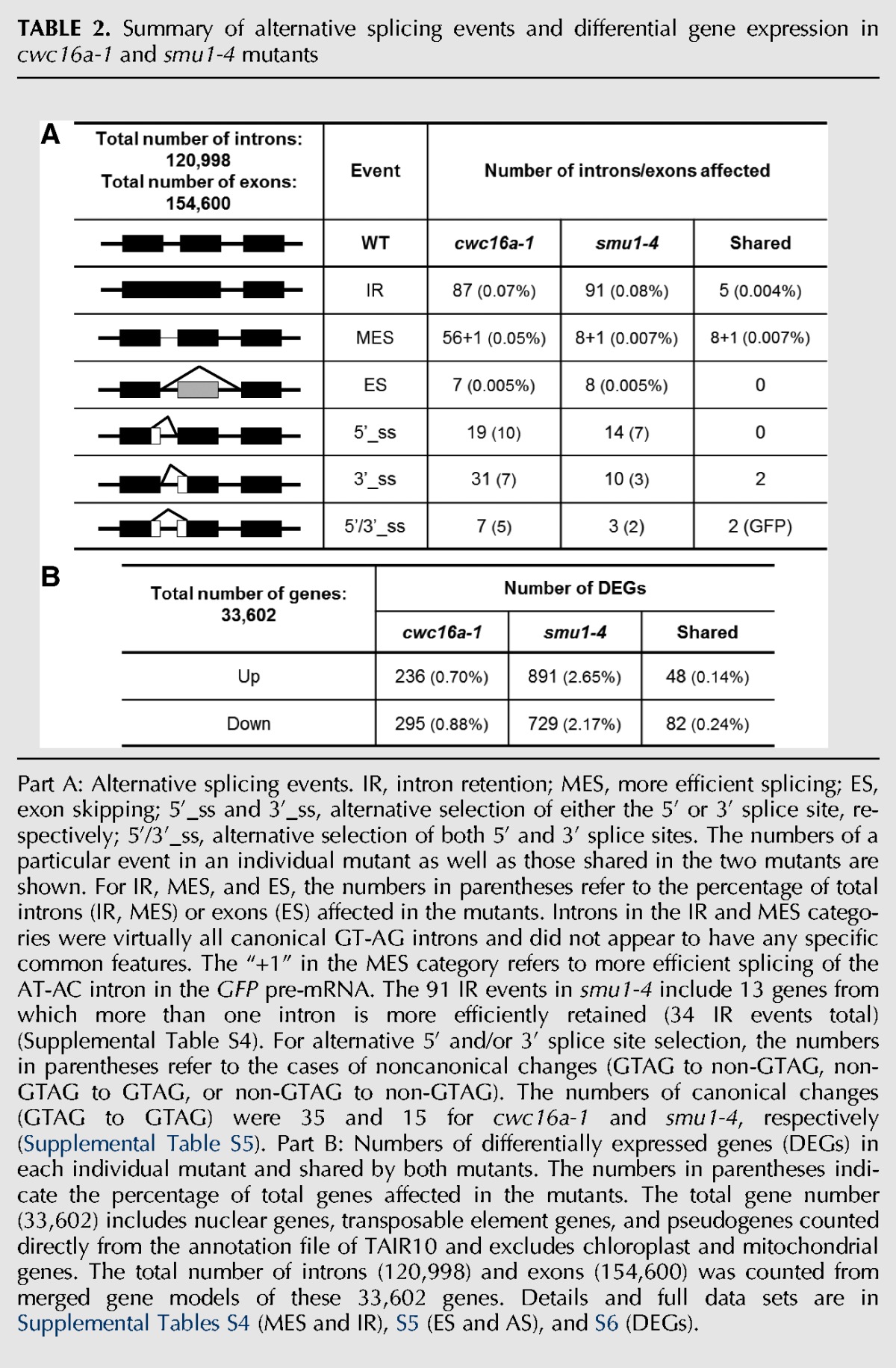
Summary of alternative splicing events and differential gene expression in *cwc16a-1* and *smu1-4* mutants

Cases of exon skipping and 5′ and 3′ alternative splice site selection were also observed in endogenous pre-mRNAs in the *cwc16a-1* and *smu1-4*. The numbers of events were relatively low in both mutants, and there were few instances of shared genes in these categories (Table 2A; Supplemental Table S5). For both *cwc16a-1* and *smu1-4,* examples of alternative splice site selection in multiple introns within a given gene were detected (Supplemental Table S5). The number of differentially expressed genes (DEGs) numbered in the hundreds for each mutant (Table 2B; Supplemental Table S6). There was a low overlap between DEGs and genes affected by alternative splicing events (Supplemental Table S7).

## DISCUSSION

In a forward genetic screen based on an alternatively spliced *GFP* reporter gene in *Arabidopsis*, we identified two factors, SMU1 and CWC16a, which influence splice site selection in *GFP* pre-mRNA. In *smu1-4* and *cwc16a-1* mutants, splicing of a U2-type intron with noncanonical AT–AC splice sites was favored over splicing of a canonical GT–AG intron. This led to increased levels of translatable *GFP* pre-mRNA and a Hyper-GFP phenotype relative to wild-type plants. A third mutant retrieved in the screen, *smfa-1*, exhibited a Hyper-GFP phenotype in the absence of substantial alterations in splicing of *GFP* pre-mRNA.

The specific roles of SMU1 and CWC16a in *GFP* pre-mRNA splicing such that the respective mutations were retrieved in our screen are unclear. The WD40-repeat protein Smu1 was originally found to regulate alternative splicing of unc-52 pre-mRNA in *Caenorhabditis elegans* (Spike et al. 2001). Although highly conserved in plants and metazoans, Smu1 is absent from budding yeast, suggesting its function is associated with complex splicing patterns (Ulrich et al. 2016). Human Smu1 has been found to interact with CUL4B-DDB1 ubiquitin E3 ligase complexes in vivo (Higa et al. 2006), indicating a role in ubiquitin-based regulation of the spliceosome. Because CUL4-DDB1 ligases use WD40-repeat proteins as adaptors for substrate recognition, it has been suggested that Smu1 may be involved in recognizing spliceosomal targets for ubiquitination (Higa et al. 2006; Chung et al. 2009). Whether SMU1 has this activity in *Arabidopsis* remains to be investigated. Several *Arabidopsis* splicing factors acquire ubiquitination, including the catalytic site core protein PRP8, which was identified among the GFP-weak mutants in our screens (Sasaki et al. 2015; this study). Homozygous *smu1-1 and smu1-2* T-DNA insertion mutants of *Arabidopsis* were reported to be nonviable or show multiple, nonlethal developmental defects, respectively (Chung et al. 2009). In contrast, the *smu1-4* mutation we identified did not result in strong growth or reproductive abnormalities. Although *smu1-4* may not be a complete loss-of-function allele, it should be noted that *smu1* null mutations in *C. elegans* are also viable and do not show an obvious phenotype (Spike et al. 2001).

The CWC16 family protein in budding yeast, Yju2, was first reported as a novel essential gene on yeast chromosome X (Forrová et al. 1992) and later shown to be a splicing factor acting at the first catalytic step of splicing in budding yeast (Liu et al. 2007; Chiang and Cheng 2013). Although Yju2 is the sole CWC16 family member in budding yeast, most eukaryotes have representatives of two classes of CWC16 protein based on the human proteins CCDC94 and CCDC130. In our screen, we identified CWC16a, which is a member of the CCDC130 class whereas yeast Yju2 is in the CCDC94 class. Even though the *cwc16a-1* mutation we identified is likely a null, the *cwc16a-1* mutant in *Arabidopsis* is viable and fertile. The CCDC94-type gene in *Arabidopsis*, CWC16b, may functionally compensate to some extent for loss of CWC16a. Alternatively, CWC16a may have a less essential and more specialized role than CWC16b in splicing. Interestingly, the “dramatically reduced” spliceosome in the red alga *Cyanidioschyzon merolae* contains an ortholog of Yju2/CWC16b/CCDC94 but not Smu1 (Hudson et al. 2015; Stark et al. 2015). This may suggest a core spliceosomal function for Yju2/CWC16b/CCDC94 and more advanced regulatory roles for CWC16a/CCDC130 and Smu1 in alternative splicing in higher eukaryotes.

### Mechanistic aspects of SMU1 and CWC16a activity in splicing

The contributions of SMU1 and CWC16a to the step-wise mechanism of pre-mRNA splicing in plants are unknown. All mechanistic information available so far on Smu1 and Cwc16 family proteins comes from other systems. In human cells, Smu1 associates transiently with the spliceosome. It is most abundant in the B complex during its transition to the catalytically active complex B* and then is released during catalytic activation (Bessonov et al. 2008; Agafonov et al. 2011; Papasaikas et al. 2015; Ulrich et al. 2016). In budding yeast, Yju2 is associated with PRP8 in the catalytic center of the spliceosome, where it promotes the first catalytic step of splicing (Galej et al. 2016; Wan et al. 2016). Based on this information, it is reasonable to predict that in *Arabidopsis*, SMU1 and CWC16a will also act, respectively, directly prior to and during the first catalytic step of splicing. For *GFP* pre-mRNA, splicing per se does not seem to be impaired in *smu1-4* and *cwc16a-1* mutants but rather splice site selection is altered: The AT–AC intron is more efficiently spliced than the GT–AG intron in the two mutants compared to wild-type plants. In *Arabidopsis*, SMU1 and CWC16a are thus able to influence intron choice, at least in some cases. Perhaps AT–AC represents a set of weaker splice sites that are used when controls on splice site selection are relaxed, which conceivably occurs in the *smu1-4* and *cwc16a-1* mutants. In contrast to *smu1-4* and *cwc16a-1*, the two GFP-weak mutants reported here, *prp8-10* and *rtf2-4* showed generally reduced splicing efficiency of *GFP* pre-mRNA, leading to increased accumulation of the unspliced primary transcript. The overall reduced splicing efficiency in these mutants is consistent with core spliceosomal roles of PRP8 (Pleiss et al. 2007) and possibly RTF2 (Larson et al. 2016), although a regulatory role through ubiquitination has also been suggested for RTF2 (Sasaki et al. 2015).

### Effects of cwc16a-1 and smu1-4 mutations on splicing genome-wide

Alternative splicing of pre-mRNAs from a modest number of endogenous genes was altered in the *smu1-4* and *cwc16a-1* mutants. The relatively small set of affected genes might reflect partial redundancy (*cwc16a-1*) or incomplete loss-of-function (*smu1-4*). Although the *cwc16a-1* and *smu1-4* mutations both alter splicing of *GFP* pre-mRNA in a similar manner, each mutant had a different effect on splicing genome-wide and a generally unique set of target transcripts. In *cwc16a-1*, roughly similar numbers of MES and IR events were observed and there were no genes in which splicing of multiple introns was affected. This finding suggests that CWC16a can either positively or negatively modulate splicing of individual introns. In contrast, the *smu1-4* mutant displayed considerably more IR than MES events, and for a notable number of genes in the first category, more than one intron was retained in the pre-mRNA. These results suggest that SMU1 is important for efficient splicing of multiple introns within a given gene. Other alternative splicing events (ES and 5′ and/or 3′ alternative splice site selection) were found to affect a relatively low number of genes, with negligible overlap between the two mutants. Examples of alternative 5′ or 3′ splice selection affecting multiple introns within a given pre-mRNA were observed for both mutants, a result that differs from the exclusive occurrence of multiple retained introns in *smu1-4*. Similar transcript specificity of pre-mRNA splicing has been observed previously for mutants of different core spliceosomal proteins in budding yeast, suggesting a complex relationship between the composition of the spliceosome and its full range of substrate RNAs (Pleiss et al. 2007). Further work is needed to understand these complicated connections and their implications for splicing efficiency and regulation.

### SMFa

SmF is a core protein of spliceosomal snRNPs and hence required at all steps of the splicing process (Matera and Wang 2014). SmF joins six other Sm proteins—B/B′, D1, D2, D3, E, and G—in a heptameric ring that encircles the snRNA moiety of the respective snRNPs, which direct pre-mRNA splicing. In *Arabidopsi*s, all seven Sm proteins are encoded by duplicated genes, only a few of which have been functionally characterized (Cao et al. 2011). Knockout mutations of SmD3-b have previously been found to alter splicing and produce pleiotropic phenotypes in *Arabidopsis* (Swaraz et al. 2011). Sm proteins can also have functions in RNA metabolism beyond splicing of primary transcripts (Lu et al. 2014; Xiong et al. 2015). In *Arabidopsis*, SmD1 participates in pre-mRNA splicing, RNA quality control, and post-transcriptional gene silencing (Elvira-Matelot et al. 2016).

It is not clear how the *smfa-1* mutation we identified leads to a Hyper-GFP phenotype. Unlike the *smu1-4* and *cwc16a-1* mutants, the ratio of the three *GFP* splice variants is not substantially altered in the *smfa-1* mutant. Therefore, obvious splicing defects of *GFP* pre-mRNA are not responsible for the observed Hyper-GFP phenotype of the *smfa-1* mutant, which grows and reproduces normally. It is possible that SmFb compensates for the loss, or partial loss, of SmFa function in spliceosomal snRNPs. Further work is required to understand the role of SmFa in modulating expression of the *GFP* reporter gene, which could conceivably also involve mRNA transport or translational regulation, and the effects of the *smfa-1* mutation on splicing genome-wide. Notably, SmFa is strongly induced by hypoxia (Cao et al. 2011), suggesting it may be functionally specialized to act as a stress-responsive gene.

### Outlook

Following early genetic studies to identify components required for constitutive splicing in budding yeast (e.g., Vijayraghavan et al. 1989), genetic screens in *Arabidopsis* and fission yeast are currently proving useful for dissecting more complex splicing pathways (Sasaki et al. 2015; Fair and Pleiss 2016; Kanno et al. 2016 and this study). Given the evolutionary conservation of many core and auxiliary spliceosomal proteins, knowledge gained from these model organisms could potentially be valuable therapeutically, since many human disease-causing mutations result in dysregulation of splicing (Douglas and Wood 2011; Scotti and Swanson 2015). Identification of additional mutants in our forward screen in *Arabidopsis* will increase functional knowledge of plant splicing factors and may suggest strategies for manipulating co- or post-transcriptional processes to optimize crop plant performance.

## MATERIALS AND METHODS

### Plant materials and forward genetic screen

For this study, we used a transgenic *Arabidopsis* line (ecotype Columbia, Col) that is homozygous for a target (*T*) locus containing an alternatively spliced *GFP* reporter gene. The *GFP* reporter gene is expressed primarily in the shoot and root apices and in the hypocotyl (stem) of young seedlings (Kanno et al. 2008; Sasaki et al. 2015). The *GFP* reporter gene has remained stably expressed at an intermediate level in the T line for ∼10 yr and is thus suitable for use in forward genetic screens. The intermediate level of *GFP* expression is unlikely to be due to partial gene silencing (Kanno et al. 2016), supporting the hypothesis that moderate levels of *GFP* translatable mRNA in the T line are maintained by a balanced ratio of alternatively spliced transcripts (Fig. 1). For simplicity, the nonmutagenized T line is referred to as “wild type” in this paper.

To perform a forward genetic screen to identify splicing factors, ∼40,000 seeds (M1 generation) of the wild-type T line were treated with the chemical mutagen EMS following a standard protocol (Kim et al. 2006). The M1 seeds were germinated on soil and the resulting M1 plants were allowed to flower and self-fertilize to produce M2 seeds, which represent the first generation when recessive mutations can be homozygous and display a phenotype. Around 280,000 1- to 2-wk-old M2 seedlings (∼seven M2 progeny per M1 plant) (Haughn and Somerville 1990) grown axenically on solid Murashige and Skoog (MS) medium in square petri dishes were screened for GFP fluorescence using a Leica M165FC fluorescence stereo microscope. Seedlings exhibiting either a GFP-weak (gfw) or Hyper-GFP (hgf) phenotype were among those selected for further investigation, which included sequencing the *GFP* reporter gene to determine whether the *GFP* coding and upstream regions contained any mutations. Plants that passed this check were considered putative *gfw* or *hgf* mutants. The present study focuses on *hgf2-1*, *hgf3-1*, and *hgf4-1* mutants.

### Next-generation mapping

Next-generation mapping (NGM) using backcrossed populations was used to determine the causal mutation in *hgf2-1*, *hgf3-1*, and *hgf4-1* mutants according to a previously published protocol (James et al. 2013). For this procedure, a given *hgf* mutant was backcrossed to the wild-type T line to produce BC1 plants, which were allowed to self-fertilize to produce BC1F2 seeds. The BC1F2 seeds were sterilized and sown on solid MS medium. BC1F2 seedlings displaying a Hyper-GFP phenotype were chosen for DNA isolation. Pooled DNA was prepared from at least 50 Hyper-GFP BC1F2 seedlings and used for sequencing on an Illumina platform. The single-nucleotide polymorphisms (SNPs) between wild-type T line and *hgf* mutants were detected by CLC Genomics Workbench 6 software (QIAGEN) to identify candidate genes.

### DNA sequence analysis of CWC16a, SMU1, and SmFa genes

Primers used for sequencing the *CWC16a* (At1g225682), *SMU1* (At1g73720), and *SMFa* (At4g30220) genes are shown in Supplemental Table S8.

### Complementation test

Complementation constructs for the *hgf* mutants were assembled using the wild-type coding sequence (CDS) and endogenous promoter and transcription terminator sequences including 5′ and 3′ untranslated regions (UTRs) (http://www.arabidopsis.org/). For *CWC16a* (At1g25682), the 930 nucleotide (nt) CDS was fused in frame to monomeric red fluorescent protein followed by the *rbcS3C* transcription terminator (Benfey et al. 1989); the endogenous promoter/5′-UTR sequence contained 1018 base pairs (bp) upstream of the ATG start codon. For *SMU1* (At1g73720), the 1536 CDS was flanked by the endogenous promoter/5′-UTR sequence, which contained 893 bp upstream of the ATG start codon, and the transcription terminator/3′-UTR sequence comprising 308 bp downstream from the translation termination codon. For *SMFa* (At4g30220), the 291 nt CDS was flanked by the endogenous promoter/5′-UTR sequence, which contained 1001 bp upstream of the ATG start codon, and the transcription terminator/3′-UTR sequence comprising 500 bp downstream from the translation termination codon.

Constructs encoding CWC16a, SMU1, and SmFa were inserted into binary vector pPZP221, which encodes resistance to gentamicin (Hajdukiewicz et al. 1994). The modified binary vector was introduced into *Agrobacterium tumefaciens* strain ASE, which was used to transform the respective *hgf2-1*, *hgf3-1*, and *hgf4-1* mutants using the floral dip method (Clough and Bent 1998). T1 seedlings were selected on solid MS medium containing gentamicin and transferred later to soil. T2 seeds resulting from self-fertilization of T1 plants were sown on gentamicin-containing MS medium and scored for segregation of gentamicin resistance and GFP fluorescence. Complementation of the Hyper-GFP phenotype resulting from *hgf2-1, hgf3-1*, and *hgf4-1* mutations was considered successful if the level of GFP fluorescence in gentamicin-resistant seedlings was restored to the intermediate level similar to that observed in the wild-type T line.

To determine whether the *hgf* mutations caused any aberrant phenotypes, we obtained BC1F2 progeny homozygous for the respective *hgf* mutations by backcrossing mutants to the T line and then allowing self-fertilization of the resulting BC1 plants to produce BC1F2 progeny. BC1F2 progeny with a Hyper-GFP phenotype were confirmed to be homozygous for the respective *hgf* mutation by DNA sequencing. For all three mutants, the BC1F2 progeny containing homozygous *hgf* mutations were viable and fertile under standard conditions on soil in a plant growth room (16 h light/8 h dark cycle, 23°C–24°C, and ∼50% humidity).

### Western blots

GFP protein was detected by Western blotting using protein extracts isolated from 2-wk-old mutant and wild-type seedlings according to a previously published protocol (Fu et al. 2015).

### RT-PCR of GFP RNAs

Semi-quantitative RT-PCR to detect *GFP* RNAs was carried out using total RNA isolated from 2-wk-old seedlings according to a protocol published previously (Sasaki et al. 2015). GFP and actin primers are shown in Supplemental Table S8.

### RNA-seq

Total RNA was isolated from 2-wk-old seedlings of the wild-type T line and the *hgf2-1/cwc16a-1* and *hgf3-1/smu1-4* mutants. Library preparation and RNA-seq were carried out (biological quintuplicates for each sample) as described previously (Sasaki et al. 2015; Kanno et al. 2016). RNA-seq reads were mapped in two steps. For the first steps, reads were mapped to the TAIR10 transcriptome using Bowtie2 (Langmead and Salzberg 2012). Only read pairs that had both been mapped to the same transcript(s) were retained and their alignments were translated to the TAIR10 genome. In the second step, the rest of the reads were mapped to the TAIR10 genome using BLAT (Kent 2002) using a default setting. Only the best alignments with identity of no less than 90% were accepted for computation, and more than 95% of the reads were accepted for every replicate (see Supplemental Table S9 for mapping statistics). RackJ (http://rackj.sourceforge.net/) was then used to compute read counts for all genes, the average depths of all exons and all introns, and read counts for all splicing junctions.

Read counts of all samples were normalized using the TMM method (Robinson and Oshlack 2010) and transformed into logCPM (log counts per million) using the voom method (Law et al. 2014) with parameter normalize = “none”. Adjusted RPKM values were computed based on logCPMs and used for *t*-tests. In this study, a gene was defined as differentially expressed if its *P*-value by *t*-test was less than 0.01, and its absolute log fold-change was greater than or equal to 0.6.

The preference of intron retention events was measured using a χ^2^ test for goodness-of-fit (Sasaki et al. 2015), in which read depths of an intron in two samples were compared to the background of read depths of neighboring exons. In this approach, the underlying null hypothesis assumes that the chances for an intron to be retained are the same in the two samples; a significant *P*-value indicates that the chance of intron retention was altered in one of the two samples. Given an intron with *P*-value less than 0.01 in all biological replicates, it was defined as more efficiently spliced if the ratio intron_depth/exon_depth in the mutant was smaller than that in the wild-type control; otherwise, it was defined as a case of increased intron retention.

The preference of exon skipping events and alternative 5′/3′ splice site selection events were measured using similar methods as those for intron retention events. For exon skipping events, splice-read counts that supported an exon-skipping event were compared to those involving a skipped exon using the χ^2^ test for goodness of fit. For alternative 5′/3′ splice site selection events, splice-read counts that supported a splicing junction were compared to those supporting other junctions of the same intron using Fisher's exact test, and the former counts were also compared to unique read counts of the same gene for further confirmation using Fisher's exact test. Here, an alternative splicing event was reported if its *P*-values were all less than 0.01 in all biological replicates, and it was defined as enhanced if the ratio of supporting read count to unique read count of the gene in the mutant is greater than that in the wild-type control; otherwise, it was defined as reduced.

### Data availability

Seeds of the wild-type T line are available at the Arabidopsis Biological Resource Center (ABRC), Ohio State University, under the stock number CS69640. Seeds of the homozygous *hgf2-1/cwc16a-1*, *hgf3-1/smu1-4*, and *hgf4-1/smfa-1* mutants will be deposited at the ABRC and are currently available on request from the Matzke laboratory. RNA sequencing data are available from NCBI SRA under accession number SRP093582.

## SUPPLEMENTAL MATERIAL

Supplemental material is available for this article.

## Supplementary Material

Supplemental Material
