# Supplementary material for: A genetic screen implicates a CWC16/Yju2/CCDC130 protein and SMU1 in alternative splicing in Arabidopsis thaliana
Source: RNA. 2017 Jul;23(7):1068–79. doi: 10.1261/rna.060517.116 (PMC5473141; doi:10.1261/rna.060517.116)
Supplement: Supplemental Material [file supp_23_7_1068__index.html]

A genetic screen implicates a CWC16/Yju2/CCDC130 protein and SMU1 in alternative splicing in Arabidopsis thaliana — Supplemental Material 

# A genetic screen implicates a CWC16/Yju2/CCDC130 protein and SMU1 in alternative splicing in *Arabidopsis thaliana*

## Supplemental Material

- Supplemental\_Fig\_S1\_rtf2\_prp8\_new\_allelesb.pdf
- Supplemental\_Fig\_S2.pptx
- Supplemental\_Fig\_S3\_CWC16a\_alignments\_plant\_.rtf
- Supplemental\_Fig\_S4\_\_CWC16\_alignments\_model\_organisms.rtf
- Supplemental\_Fig\_S5\_CWC16\_alignments\_Arabidopsis\_thaliana.rtf
- Supplemental\_Legends.docx
- Supplemental\_Table\_S1\_CWC16\_family\_orthologs\_ccdc\_94\_or\_130.xlsx
- Supplemental\_Table\_S2\_GFP\_nptII\_transcription\_not\_significantly\_altered.xlsx
- Supplemental\_Table\_S3\_gfpDepth.xlsx
- Supplemental\_Table\_S4\_MES\_IR\_210217.xlsx
- Supplemental\_Table\_S5\_AS\_ES\_.xlsx
- Supplemental\_Table\_S6\_DEGs\_.xlsx
- Supplemental\_Table\_S7\_DEGs\_alternative\_splicing\_overlap.xlsx
- Supplemental\_Table\_S8\_primers.docx
- Supplemental\_Table\_S9\_RNA\_read\_mapping\_statistics.xlsx
