# Supplementary material for: A genetic screen implicates a CWC16/Yju2/CCDC130 protein and SMU1 in alternative splicing in Arabidopsis thaliana
Source: RNA. 2017 Jul;23(7):1068–79. doi: 10.1261/rna.060517.116 (PMC5473141; doi:10.1261/rna.060517.116)
Supplement: Supplemental Material [file supp_060517.116_Supplemental_Fig_S1_rtf2_prp8_new_allelesb.pdf]

Kanno et al.   Supplemental Figure S1

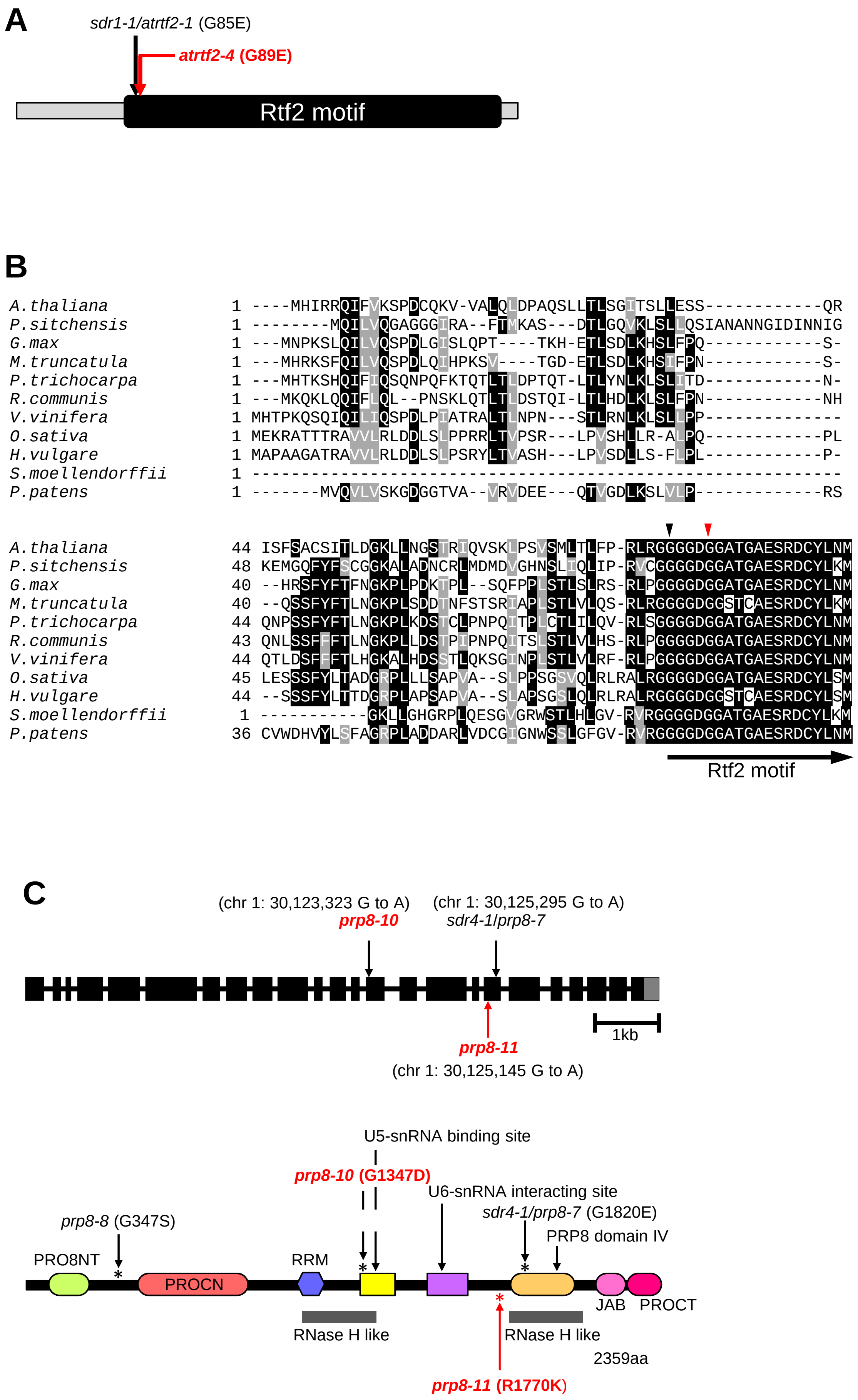

Supplemental Figure S1: *gfw1* and *gfw2* are new alleles of *rf2* and *prp8*

In previous work, we identified *rtf2-3* (At5g58020) and *prp8-7* (At1g80070) as mutant alleles conferring a GFP-weak (*gfw*) phenotype in a previous screen based on the same alternatively spliced *GFP* reporter gene used in this study (Sasaki et al., 2015). In the present screen, we identified additional new alleles of *rtf2/gfw1* and *prp8/gfw2* based on their GFP-weak phenotypes, which is likely due to increased proportions of unspliced untranslatable *GFP* transcript (**Figure 4**). RTF2 is increasingly recognized as an important splicing-related protein and has been identified not only in our forward genetic screens but also in a recent sequence-based genetic screen conducted in fission yeast to identify splicing factors (Larson et al., 2016). PRP8 is one of the largest and most highly conserved spliceosomal proteins and is situated at the catalytic center of the spliceosome (Grainger and Beggs, 2005).

A and B: The new mutant allele of *rtf2* we identified in the present study, *rtf2-4*, creates a G89E substitution in the highly conserved Rtf2 motif of the RTF2 protein (**A**). This is in close proximity to the previous mutant allele we identified, *sdr1-1*/*rtf2-1*, which causes a G85E substitution (**A**). These apposed glycine residues are in a short G-rich region at the beginning of the Rtf2 domain (**B, red arrowheads**). The positions of the two mutated residues suggest that the run of glycines is critical for the function of RTF2 in pre-mRNA splicing. Details of its role in the mechanism of splicing remain to be investigated.

C: We identified two new *prp8* alleles in the present screen: *prp8-10* and *prp8-11*. The mutant *prp8-10* allele causes a G1347D substitution at the edge of the U5-snRNA binding site (yellow box). The mutant *prp8-11* allele causes a R1170K substitution. The mutant allele we identified in the previous screen (*sdr4-1/prp8-7*) creates a G1820E substitution in the second RNase H-like domain (Sasaki et al., 2015).

Grainger RJ, Beggs JD. 2005 Prp8 protein: at the heart of the spliceosome. *RNA* **1**: 533-557

Larson A, Fair BJ, Pleiss JA. 2016. Interconnections between RNA-processing pathways revealed by a sequencing-based genetic screen for pre-mRNA splicing mutants in fission yeast. *G3* (Bethesda) **6**: 1513-1523

Sasaki T, Kanno T, Liang SC, Chen PY, Liao WW, Lin WD, Matzke AJ, Matzke M. 2015.
An Rtf2 domain-containing protein influences pre-mRNA splicing and is essential for embryonic development in *Arabidopsis thaliana*. *Genetics* **200**: 523-535
