# Supplementary figures and images for: A genetic screen implicates a CWC16/Yju2/CCDC130 protein and SMU1 in alternative splicing in Arabidopsis thaliana
Source: RNA. 2017 Jul;23(7):1068–79. doi: 10.1261/rna.060517.116 (PMC5473141; doi:10.1261/rna.060517.116)

## Slide 1
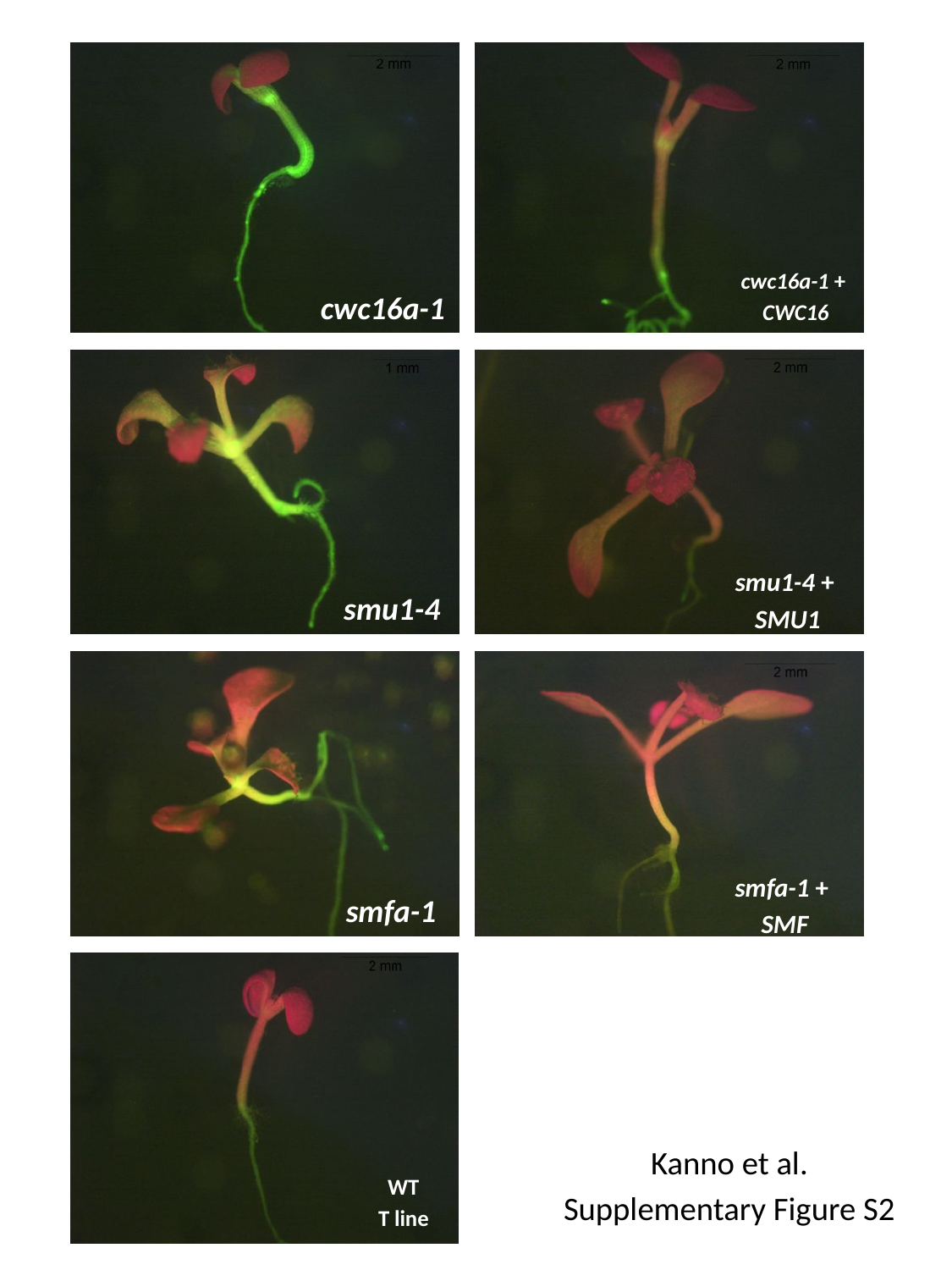

cwc16a-1 +
CWC16
cwc16a-1
smu1-4 +
SMU1
smu1-4
smfa-1 +
SMF
smfa-1
cwc16 + CWC16
Kanno et al.
Supplementary Figure S2
WT
T line

Supplement: Supplemental Material [file supp_060517.116_Supplemental_Fig_S2.pptx]
