# Supplementary material for: A genetic screen implicates a CWC16/Yju2/CCDC130 protein and SMU1 in alternative splicing in Arabidopsis thaliana
Source: RNA. 2017 Jul;23(7):1068–79. doi: 10.1261/rna.060517.116 (PMC5473141; doi:10.1261/rna.060517.116)
Supplement: Supplemental Material [file supp_060517.116_Supplemental_Fig_S3_CWC16a_alignments_plant_.rtf]

Supplementary Figure 3 Kanno et al.

S.moellendorffi	1 ----------------------------------MIDAFAVFLFQSTLAAARADNFYFPP
C.reinhardtii	1 --------------------------------------------MSSLAAARADNFYYAP
P.patens		1 -MLHGFPLRQTESFLFARIRVLSILVSEA-YFVYLRVVTYTGSTMSTLAAARADNFYYPP
A.trichopoda	1 -----------------------MCACDVGLCDYIVVDGTSLFPRSSLAAARADNFYYPP
A.thaliana		1 --------------------------------------------MSTLSAARADNFYYPP
B.rapa1		1 --------------------------------------------MSTLSAARADNFYYPP
B.rapa2		1 --------------------------------------------MSTLSAARADNFYYPP
O.sativa		1 --------------------------------------------MSSLAAARADNFYYPP
S.bicolor		1 --------------------------------------------MSSLAAARADNFYYPP
B.distachyon	1 --------------------------------------------MSTLAAARADNFYYPP
P.abies		1 --------------------------------------------MSSLAAARADNFYYPP
S.lycopersicum	1 --------------------------------------------MSSLAAARADNFYYPP
G.max1		1 --------------------------------------------MSSLAAARADNFYYPP
G.max2		1 --------------------------------------------MSSLAAARADNFYYPP
V.vinifera		1 MDWDGVTRLEPSHFILDYVAYLSKIVRARTESRASNLGKNEEDNMSSLAAARADNFYYPP
P.trichocarpa1	1 --------------------------------------------MSSLAAARADNFYYPP
P.trichocarpa2	1 ---------------MSLISFLTILTC-----------FMTMYFQSSLAAARADNFYYPP


S.moellendorffi	27 EWTPKQQGSLNKFQGQHPLRERARKIDQGILVIRFEMPFNVWCGGCHSMIAKGVRFNAEK
C.reinhardtii	17 DFDPKKHKSLNKYNGQHPLRERAAKLDQGILVIRFEVPFNIWCDKCGEHIAKGERFNAEK
P.patens		59 EWTPDQ-GGLNKFNGQHALRERAKKIDQGILVIRFEMPYHIWCGGCGHMIAQGVRFNAEK
A.trichopoda	38 EWTPSQ-GSLNKFHGQHALRERARKIDQGILIIRFEMPFNIWCGGCNAMIAKGVRFNAEK
A.thaliana		17 EWTPDQ-GSLNKFQGQHPLRERAKKIGEGILVIRFEMPYNIWCGGCSSMIAKGVRFNAEK
B.rapa1		17 EWTPEQ-GSLNKFQGQHPLRERARKLGEGILIIRFEMPYNIWCGGCNSMIAKGVRFNAEK
B.rapa2		17 EWTPDQ-GSLNKFQGQHPLRERAKKIGEGILIIRFEMPYNIWCGGCNSMIAKGVRFNAEK
O.sativa		17 EWSPKK-GGLNKFHGQHALRERARKLDQGILIIRFEMPFNIWCGGCNSMIAKGVRFNAEK
S.bicolor 		17 EWSPKK-GGLNKFHGQHALRERARKLDQGILIIRFEMPFNIWCGGCNSMIAKGVRFNAEK
B.distachyon	17 EWSPKK-GGLNKFHGQHALRERARKLDQGILVIRFEMPFNVWCGGCNSMIGKGVRFNAEK
P.abies		17 EWTPQQ-GSLNKFHGQHALRERARKIDQGILIIRFEMPFNIWCGGCQSMIAKGVRFNAEK
S.lycopersicum	17 EWSPKK-GSLNKFRGQHALRERARKIDQGILIIRFEMPFNIWCGGCESMIAKGVRFNAEK
G.max1		17 EWEPSQ-GSLNKFHGQHALRERARKLDQGILIIRFEMPFNIWCGGCNSMIAKGVRFNAEK
G.max2		17 EWEPNQ-GSLNKFHGQHALRERARKLDQGILIIRFEMPFNIWCGGCNSMIAKGVRFNAEK
V.vinifera		61 EWTPNQ-GSLNKFHGQHALRERARKIDQGILIIRFEMPFNIWCGGCNSMIAKGVRFNAEK
P.trichocarpa1	17 EWSPKK-GGLNKFHGQHALRERARKLDQGILIIRFEMPFNIWCGGCNSMIAKGVRFNAEK
P.trichocarpa2	35 EWSPKK-GGLNKFHGQHALRERARKLDQGILIIRFEMPFNIWCGGCNSMIAKGVRFNAEK


S.moellendorffi	 87 KQIGKYYTTKIWSFSMKAACCKHEIEIHTDPKNCEYVIVKGAERKTETYDAADAGSLVVA
C.reinhardtii	 77 KAIGNYHSTKILQFSMTHH-CGCTICIQTDPKNAEYIVVEGARKK---------------
P.patens		118 KQVGNYYSTKIWSFKMKAPCCGQEIVIQTDPKNTLYVIISGAKEKTVTYDEVDAEAVLLP
A.trichopoda	 97 KQVGNYYSTKIWSFTMKSACCKHEMVIQTDPQNCEYVIISGAERKTEVFDVEDAETLELP
A.thaliana		 76 KQVGNYYSTKIWSFAMKSPCCKHEIVIQTDPQNCEYVITSGAQKKVEEYEAEDAETMELT
B.rapa1		 76 KQVGNYYSTKIWSFAMKAPCCKQEIVIQTDPQNCEYVITSGAQKKVEEYDVEDAETMELT
B.rapa2		 76 KQVGNYYSTKIWSFAMKAPCCKQEIVIQTDPQNCEYVITSGAQKKVEEYDVEDAETMELT
O.sativa		 76 KQVGNYYSTKIWSFTMKSPCCKQEIVIQTDPKNTEYVIISGAQRKTEDYDVEDAETLLLP
S.bicolor		 76 KQVGNYYSTKIWSFTMKSPCCKHEIVIQTDPKNTEYVIISGAQKKTEDFDVEDAETLLLP
B.distachyon	 76 KQVGNYYSTKIWSFTMKSPCCQHEIVIHTDPKNTEYVIISGAQRKTEDFDVEDAETLLLP
P.abies		 76 KQVGNYYSTKIWSFSMKAACCRQEIVIHTDPKNCEYVIISGAERKNEEFDAEDAETLALP
S.lycopersicum	 76 KQVGNYYSTKIWSFSMKSACCKHEIVIQTDPKNCAYVIISGAQKKTEDYDAEDAETLVLP
G.max1		 76 KQVGNYYSTKIWSFAMKSACCKHEIVIQTNPKNCEYVIISGAQKKTEDFDIEDAETFELP
G.max2		 76 KQVGNYYSTKIWSFTMKSACCKQEIVIQTDPKNCEYVIISGAQKKTEDFDIEDAETFELP
V.vinifera		120 KQVGNYYSTKIWSFTMKSACCKHEIVIQTDPKNCEYVIISGAQRKTEEFDIEDAETFALP
P.trichocarpa1	 76 KQVGNYYSTKIWSFTMKSACCKHEIVIQTDPKNCEYVIISGAQRKNEEFDIEDAETFALP
P.trichocarpa2	 94 KQVGNYYSTKIWSFTMKSACCKHEIVIHTDPKNCEYVIISGAQRKNEEFDVEDAETFALP


S.moellendorffi	147 TGEDREKLTDPFAKLEHDEEDILKGKESAKQLMLLQKQSDIKHGNDYSVNRALRAQLRDQ
C.reinhardtii           ------------------------------------------------------------
P.patens		178 EKEDRGKLADPFYKLEHEGEDTAKAKKQAPLLVRLQEAADRKHSDSYARNKALRAQLRAQ
A.trichopoda	157 ADEERGKLADPFYRLEHQEADLQKKKEADPLLVRLQRASDVKHANDYHLNRTLRAQIRSQ
A.thaliana		136 AEQEKGKLADPFYRLEHQEVDLQKKKAAEPLLVRLQRVSDARHADDYSLNKALRAQLRRH
B.rapa1		136 AEEEKGKLADPFYRLEHQEVDLQKKKAAEPLLVRLQRVSDARHADDYSLNKALRAQIRGH
B.rapa2		136 AEEEKGKLADPFYRLEHQEVDLQKKKAAEPLLVRLQRVSDARHADDYSLNKALRAQIRGQ
O.sativa		136 ADEERDKLADPMYKLEHQEEDLKKKKEAEPVLVRLQRLSDSRHSDDYALNRALRDRLRSQ
S.bicolor		136 ADEDRDKLADPMYRLEHQEEDLRKKKEAEPVLVRLQRLSDSRHSDDYALNRALRDRLRSQ
B.distachyon	136 ADEERDKLADPMYKLEHQEEDIRKKKEEEPVLVRLQRLSDSRHSDDYALNRALRDRLRSQ
P.abies		136 ADEERGKLADPFYRLEHEGEDLQKGKEAAPFLVRLQRSSDLKHADDYSRNRALRAEVRSQ
S.lycopersicum	136 VDEDKSKLVDPFYRLEHQEEDLKKKKKAEPLLVRLQRVSDTRHSDDYAMNKALRATLRGQ
G.max1		136 ADEERGKLADPFYRLEHQEEDLKKKKEAEPVIVRLQRQSDARHSDDYYLNKTLRAQLRSQ
G.max2		136 ADEEKGKLADPFYRLEHQEEDLKKKKEAEPVIVRLQRQSDARHSDDYSLNKTLRAQLRGQ
V.vinifera		180 ADEERGKLADPFYRLEHEGEDLQKKKEAEPVLVRLQRVSDARHSDDYSLNKALRAQLRNQ
P.trichocarpa1	136 ADEERGKLADPFYRLEHQEEDLQKKKEAEPILVRLQRVSDARHLDDYSLNKALRAQMRSQ
P.trichocarpa2	154 ADEEKGKLSDPFYRLEHQEEDLQKKKEAEPVLVRLQRVSDARHSDDYALNKALRARMRSH


S.moellendorffi	207 KKRVALEEAHSKKLGIGIRLLPASEDDAQAAGSVRFAQKFDKNRRDKRAAIRATSIFSQS
C.reinhardtii        ------------------------------------------------------------
P.patens		238 KKRVAAEEVEAQKLGLAIRLLPPSKEDSDYAANVKFASNFGLNQRNKRAAIQATSIFSSE
A.trichopoda	217 RKRVAEEEATARKMGLGIRLLPASKEDSDVAASVKFAPQFDRNKRERRDAINNQPLFSKS
A.thaliana		196 RKRVAEEETASRKLGLGIRLLPKSEEDIKAASNVKFKSKFDKNRKDKRALIHASSIFPES
B.rapa1		196 RKRVAEEEAASRKLGLGIRLLPKSEEDIAAASNVKFKTKFDKNRKDKRALIHASSIFPES
B.rapa2		196 RKRVAEEEAASRKLGLGIRLLPKSEEDVAAASHVKFKTKFDKNRKDKRALIHASSIFPES
O.sativa		196 KKRVAEEKRSARKMGLGVRLLPPSAEDATAAASVKFASKFEKSRRDKRAAIKAASIFPES
S.bicolor		196 KKRVAEEKKSARKMGLGVRLLPPSAADAAAAASVKFASRFEKNRKDKRAAIKAASIFPES
B.distachyon	196 KKRVAEEKKSARKMGLGVRLLPPSAEDAAAAASVKFASKFEKSRKDKRAAIKASSIFPES
P.abies		196 KKRVAEETAHSRKRGLGIRLLPASKEDTEMAAKIKFSSRFEMNRRDKRAAINASSIFSDS
S.lycopersicum	196 KKRVAEEEAAAKKVGLGIRLLPPSTEDAATAASVKFAHKFDKNRRDKRAMIYSGSIFGSS
G.max1		196 KKRVTEEENASKKRGLGIRLLPATEQDSATAKSVKFSAKFDKNRKDKRALISSESIFSGV
G.max2		196 KKRVAQEENASKKRGLGIRLLPATEQDSATAKSVKFPAKFERNRKDKRALINSESIFSGV
V.vinifera		240 KKRVAEEEFVSKKLGLGIRLLPATEEDAAIAARMKFSSKFERNRKEKRALINAASIFPGS
P.trichocarpa1	196 KKRVAEEEATSKKMGLGIRLLPTTEEDGASAAHVKFSSKFDKNRKDKRALISADSIFSGS
P.trichocarpa2	214 KKRVAEEESTSRKMGLGIRLLPATEEDAVSAAHVKFSSKFDKNRKDKRALISAASIFPGS


S.moellendorffi	267 GAYG-------LPPAGSDQKLQLLAKRRKIQAAEAQSLLRGRIKASSLAAQRS*------
C.reinhardtii        ------------------------------------------------------------
P.patens		298 TSKHSTNTSRPSPASALRQKLDLLAKRRRVNAAGAKEILCSNVKPLTRGVLERGSLSVRP
A.trichopoda	277 SGSE-----------SIKRKLELMEKRRKIKASAANAMLLGSTKPSSWSRPHISRAPKR-
A.thaliana		256 SYS------------SSKKRMELEAKRRKISAASASSLLRGGFKASSLSTNPSASKP-KV
B.rapa1		256 SYSMS---------SSSKKRLELEAKRRKICAASASSLLSGGFKASSLSKNPSSANKYKS
B.rapa2		256 SYSMS---------TSSKKRLELEAKRRKICAASASSLLSGGFKASSLSKTPSSSTKFKS
O.sativa		256 SSS-T-----------SKNKLDLALKRRNIKAGAASALMASRVKPSSWQSAGSGSSRTQM
S.bicolor		256 SSS-A-----------SNCKLDLVLKRRNIKAGAATTLMAGRVKPSSWQSASSASSRTRM
B.distachyon	256 PSS-A-----------SKDKLDLALKKRNIKAGAASMLMAGRVKPSSLQSVGSRSASTHV
P.abies		256 S-Y-S---------KSTDKRLELLAKRRKINATAAHSSLCGKFKPSSISQNTSGSNIGAR
S.lycopersicum	256 GS---------------SKHSELESKRRKINASAASKLLVGGFKPSSWSEATVPSKKRRV
G.max1		256 SSY-S---------MSDKRKRELESKRRKICATSASSLLAGRVKPSSWSQPSSKQKGTVR
G.max2		256 SSY-S---------ISDKRKQELESKRRKICATSASNLLAGRVKPSSWSQSSTKQKGTMR
V.vinifera		300 SGS-S---------LSDKKRLELGSKRRKIKAGTASELLTRGFKPSSWLKSSVSSSQSRG
P.trichocarpa1	256 SGS-S---------MSNKKRLELESKRRKISASAASNLLTGGFKPSSWSQGTVSGSRHKQ
P.trichocarpa2	274 SGS-S---------MSNKKRLELESKRRKISAAAATNLLTGGFKPSSWSQGTVSCSKRKQ


S.moellendorffi      ---------
C.reinhardtii        ---------
P.patens 		358 PV-KLKQA*
A.trichopoda 	325 SIVSVRRG*
A.thaliana		303 SSVSVRKL-
B.rapa1		307 STVSVRKM*
B.rapa2		307 STVSVRKL*
O.sativa		304 PIMATRK*-
S.bicolor		304 PVLAARK*-
B.distachyon	304 RVLARRK*-
P.abies		305 ALVVVKRG*
S.lycopersicum	301 *--------
G.max1		306 C*-------
G.max2		306 R*-------
V.vinifera		350 MSVASRHL-
P.trichocarpa1	306 NSMSARLF*
P.trichocarpa2	324 NSMNARRV*


Supplementary Figure 3: Amino acid sequence alignments of CWC16a proteins in plants
The amino acid sequences of CWC16a proteins from a number of plant species are aligned. The protein sequences belong to Family: CELL CYCLE CONTROL PROTEIN CWF16-RELATED; Subfamily: COILED-COIL DOMAIN-CONTAINING PROTEIN 130 (CCDC130) (PTHR12111:SF5) in PANTHER (http://www.pantherdb.org/panther/) and/or COILED-COIL DOMAIN-CONTAINING PROTEIN 130 (PTHR12111:SF2) in Phytozome (https://phytozome.jgi.doe.gov/) in addition to the longest homologous protein to At1g25682 in P. abies (BLASTed at http://congenie.org). The sequences were aligned by Clustal Omega (http://www.ebi.ac.uk/Tools/msa/clustalo/). The alignment result was visualized by BOXSHADE (http://www.ch.embnet.org/software/BOX_form.html).
