# Supplementary material for: A genetic screen implicates a CWC16/Yju2/CCDC130 protein and SMU1 in alternative splicing in Arabidopsis thaliana
Source: RNA. 2017 Jul;23(7):1068–79. doi: 10.1261/rna.060517.116 (PMC5473141; doi:10.1261/rna.060517.116)
Supplement: Supplemental Material [file supp_060517.116_Supplemental_Fig_S4__CWC16_alignments_model_organisms.rtf]

Supplementary Figure 4 Kanno et al.

S.cerevisiae	1 ---MSERKAINKYYPPDYNPLEAEKLSRK-----MAKKLKTM-NKSHASIRLMTPFSMRC
N.crassa		1 ----MQGFNMGKYVPPDAEGVY--SGNQLSKKHPLGHRASKLASQGILTVRFELPFAVWC
S.pombe		1 ----MQGFNMGKYIPPEGPN-----------------AKRKF-DKLRNVIRFEMPFPVWC
T.thermophila	1 MSSLKAARADNYYYPQDWDPSK-GSLNKFQNSHPLGDRAKKI-DQGILVIRYETPFHVRC
A.thaliana		1 MSTLSAARADNFYYPPEWTPDQ-GSLNKFQGQHPLRERAKKI-GEGILVIRFEMPYNIWC
C.elegans		1 ---MGERKGQNFYYPPDFNYKTHKSLNGYHGTHALRERAKKI-DQGILVIRFEMPFNIWC
D.melanogaster	1 ---MGERKGQNKYYPPDYDPKK-GGLNKFQGTHALRERARKI-HLGIIIIRFEMPYNIWC
D.rerio		1 ---MGERKGVNKWYPPDFDPAKHGSINGYYKTHPLRERARKL-SQGILIIRFEMPYNIWC
X.tropicalis	1 ---MGERKGTNKYYPPDFDPAKHGSLNRYRNSHPLRERARKL-SQGILIIRFEMPYNIWC
H.sapiens		1 ---MGERKGVNKYYPPDFNPEKHGSLNRYHNSHPLRERARKL-SQGILIIRFEMPYNIWC
M.musculus		1 ---MGERKGQNKYYPPDFNPEKHGSLNRYHNSHPLRERARKL-SQGILVIRFEMPYNIWC
R.norvegicus	1 ---MGERKGQNKYYPPDFNPEKHGSLNRYHNSHPLRERARKL-SQGILIIRFEMPYNIWC

S.cerevisiae	52 LECNE---YIPKSRKFNGKKELLKEKYLDSIKIYRLTISCPRCANSIAFRTDPGNSDYVM
N.crassa		55 DHCQPHPTIIGQGVRFNAAKKKVGNYY--STPIWSFTIKHGQCGGEIEIRTDPKLTRYVV
S.pombe		39 NNCEN---IIQQGTRFNAVKKEIGSYY--TTKIWSFSLKCHLCSNPIDVHTDPKNTEYIV
T.thermophila	59 LKCDN---MIAKGVRFNAEKRKVGKFM--SSTIWEFKMRCPHCSNNIIVETDPENTDYKY
A.thaliana		59 GGCSS---MIAKGVRFNAEKKQVGNYY--STKIWSFAMKSPCCKHEIVIQTDPQNCEYVI
C.elegans		57 LGCHN---HVGMGVRYNAEKKKIGMYY--TTPLHEFRMKCHLCDNYYVIRTDPKNFDYEL
D.melanogaster	56 DGCKN---HIGMGVRYNAEKTKVGMYY--TTPVFKFRMKCHLCDNHFEIQTDPGNLDYVI
D.rerio		57 DGCKN---HIGMGVRYNAEKKKVGNYY--TTPIYRFRMKCHLCVNYIEMQTDPATCDYVI
X.tropicalis	57 DGCKN---HIGMGVRYNAEKKKVGNYY--TTPIYRFRMKCHLCV-LIEMQTDPANCDYI-
H.sapiens		57 DGCKN---HIGMGVRYNAEKKKVGNYY--TTPIYRFRMKCHLCVNYIEMQTDPANCDYVI
M.musculus		57 DGCKN---HIGMGVRYNAEKKKVGNYY--TTPIYRFRMKCHLCVNYIEMQTDPANCDYVI
R.norvegicus	57 DGCKN---HIGMGVRYNAEKKKVGNYY--TTPIYRFRMKCHLCVNYIEMQTDPANCDYVI

S.cerevisiae	109 EVGGVRNYVPQKPNDDLNAKTA------VESIDETLQRLVREKEMEQNEKMGIKEQADDK
N.crassa		113 VSGGRARDTGTDEESLVKLGLEGSAGGGFEIQTEKERQ----------------EQRDAA
S.pombe		 94 ASGGRRKIEPQDINERP--------------AKAENDE----------------KVPSDA
T.thermophila	114 KEGARRILNTENAKTDVQ-F----------IRDDSEKA----------------KIQEDA
A.thaliana		114 TSGAQKKVEEYEAEDAETME----------LTAEQEKG----------------KL-ADP
C.elegans		112 VEGCSRQELRFDPTDIAQIG----------AVDRGFTQ----------------KLAADA
D.melanogaster	111 LSGARRQENRWDPLQNEQVV----------PETKEVQK----------------RLFDDA
D.rerio		112 VSGAQRKEERWDMAENEQIL----------TTERNEKE----------------KLETDA
X.tropicalis	110 KSGAQRKEERWDMQDNEQIL----------TTEHEEKQ----------------RLETDS
H.sapiens		112 VSGAQRKEERWDMADNEQVL----------TTEHEKKQ----------------KLETDA
M.musculus		112 VSGASRKEERWDMEDNEQVL----------TTEHEKKE----------------KLETDA
R.norvegicus	112 VSGASRKEERWDMEDNEQVL----------TTEHEKKE----------------KLETDA

S.cerevisiae	163 MDLLEKRL-------------AKIQQ----EQEDDEELENLRKKNLEMSQRAEMINRSKH
N.crassa		157 FGKLEKTIADRERAEEAKVRIDELLEAQEKAWEDPYARNQKLRK--------AFRVGRKE
S.pombe		124 IEALETQLTQQKSEKHNSSVINFIYEKNERLWSDPFVSSQRLRK--------QFRERKKI
T.thermophila	147 FYNLENKNSDQKVAEIEKPRIQSMMQMSSDRYENDFDNNLLLRN--------KFRKEKKQ
A.thaliana		147 FYRLEHQEVDLQKKKAAEPLLVRLQRVSDARHADDYSLNKALRA--------QLRRHRKR
C.elegans		146 MFKKEHEAEDKDKAATEEGRVDKLEW-IQERMRDDFTANSFLRA--------QFRNEKKS
D.melanogaster	145 MYKLEHQAKDAKAGADARPVLQKLVERNMSVWDDSYMANSRLRA--------EFRQQKKE
D.rerio		146 MYKLDHGGKDKEKLRAAIPSLNELQE-HQSGWKDDFQLNSALRR--------KFRTEKKV
X.tropicalis	144 MFRLEHGAKDKAKLQRAAPSLSELQE-VQSAWKDDFAINSLLRS--------KFRDEKKQ
H.sapiens		146 MFRLEHGEADRSTLKKALPTLSHIQE-AQSAWKDDFALNSMLRR--------RFREKKKA
M.musculus		146 MFRLEHGEADRSTLKKALPTLSHIQE-AQNAWKDDFALNSMLRR--------HFREKKKA
R.norvegicus	146 MFRLEHGEADRSTLKKALPTLSHIQE-AQNAWKDDFALNSMLRR--------HFREKKKA

S.cerevisiae	206 AQQEKAVTT------------------D--DLDNLVDQVFDNHRQRTNKPGNNNDEK---
N.crassa		209 REKEAERTEDLRERMGLG-IELLPGTEEDERRARLIE--F----GGVPDVAQGRDDVVQK
S.pombe		176 EKKQEAKDLSLKNRAALD-IDILPPSSSDKDKALLLLD-N---ELGKNKFIRKLDYRRTL
T.thermophila	199 LEKKEEEDKKN---VNF-VLPIAPTRQTDMIRVNNAH--F----KGPNNYQKNKIEKRSE
A.thaliana		199 VAEEETASR----KLGLG-IRLLPKSEEDIKAASNVK--F---K---SKFDKNRKDKRAL
C.elegans		197 LNETRARDANLRDKLSIGTTQLLPETEEDRRIASMMT--R---YRDTKTHDDHLESSRDR
D.melanogaster	197 INGQQELDRQLLAKSSLD-IALLPETTQDREMAALMK--L----QTKSALERESE-QRLE
D.rerio		197 IAEEEEKDNAVRLRTGLS-IPLVPEREEDKKLASLLT--F----QSPDSYEDKKQWKRQE
X.tropicalis	195 IKEEEERDQALLKKASLD-LKLVPEKEEDKKLAALLK--Y----RSLESYEQKQKKKRSE
H.sapiens		197 IQEEEERDQALQAKASLT-IPLVPETEDDRKLAALLK--F----HTLDSYEDKQKLKRTE
M.musculus		197 MQEEEEKDQALQAKASLA-IPLVPESEDDRRLAALLR--L----HTLDSYEDKQRMKRTE
R.norvegicus	197 MQEEEEKDQALQAKANLA-IPLVPESEDDRRLAALLR--L----HTLDSYEDKQRMKRTE
S.cerevisiae	243 --RTPLFNPTSTKGKIQ----------KK-------------------------------
N.crassa		262 ALARPLFGDDGKNGNTE--EKKAGKKKDASKGKLKSEIAATKMREALVSE-IVGNTRAAK
S.pombe		231 MPSSRTFSTFAKFAETS------FAKKDPFARKFVPSEKLRSEQRKFPTENLKGEKILED
T.thermophila	249 ILRANIFQDQKQEEVNK-----VYKKIDKLPKS---------------------TQIMIK
A.thaliana		246 IHASSIFPESSYSSSKK------RMEL-------------EAKRRKISA---ASASSLLR
C.elegans		252 IESRRIFRRPEETDTPSTSSGSSGGAVPSASERLKAT--MKAERDKRIN-----------
D.melanogaster	249 LLMRPALPGATVTT--------FGGL----------------KRQKVLN-----------
D.rerio		250 ISSRSWFNSPSSAA-----GGAAGSLLQKLGQQ---------GRGAAVA-----------
X.tropicalis	248 ICNRSWFSPGADSGQ-----QTPGNALRKLGIR---------PR-TLSA-----------
H.sapiens		250 IISRSWFPSAPGSA----SSSKVSGVLKKLAQS---------RRTALAT-----------
M.musculus		250 IIHRSWFPSAQGPSA---SSSKASSVLKKLCQG---------RRPP-PS-----------
R.norvegicus	250 IIHRSWFPSAQGPST---SSSKASTVLKKLCRG---------RRPP-TG-----------

S.cerevisiae	260 SS-------------VRTNPLGIVIKRGKSLK----------------------------
N.crassa		319 DPFLDWGSRESTPKPRGALIPGLKRKRAAGE---E--TPDPPPVGAASLMT---------
S.pombe		285 NSV------------------SLVNYEVSDDEG---------------------------
T.thermophila	283 NSLK-LNQKS-------QNQFNIVHKK---------------------------------
A.thaliana		284 GGFK-ASSLSTNPSASKPKVSSVSVRKL--------------------------------
C.elegans		299 ASFS-TAGTSS----ATQKLLGIKRKSASSLGVQI-------------------------
D.melanogaster	274 ------TQL-------QVQDLGIRRKKLEETTSSATNE----------------------
D.rerio		285 ------KALSS-----STSTLPILVRRKSESSKSETNNTM--------------------
X.tropicalis	282 ------PGIS-------PVTLGVVRRTSKEENRAEDKLPASPNGSCSSSAGGESVREETG
H.sapiens		286 ------SPIT-------VGDLGIVRRRSRDV-------PESPQHAADTPKSGEPRVPEEA
M.musculus		286 ------STGT-------VGDLGIVRRKSRDV-------PESPQCAADNSLSEEQRRPPGT
R.norvegicus	286 ------SAGA-------PGDLGIVRRKSREA-------PESPQCTADNSLSEEPRGPPGT

S.cerevisiae            ------------------------------------------------------------
N.crassa		365 ---SRVAEGR--------DAEE------------------TKSGKQTTGSG--------S
S.pombe                  ------------------------------------------------------------
T.thermophila	     ------------------------------------------------------------
A.thaliana		     ------------------------------------------------------------
C.elegans		329 ---KKAAPENSS-LQDEEPAVEKPNISN--------------------------------
D.melanogaster	299 ------------------------------------------------------------
D.rerio		314 ---STILPVDTHPAAKDTDATDLPSIVNNINVSINTDINSCTTDAC---KASSSSEEENS
X.tropicalis	329 -----------------SKVESRERT---------QSESDLSS-VQTLATSTSTAQGKTS
H.sapiens		326 AQDRPMSPGDCPPETTETPKCSSPRG---------QEGSRQDKPLSPAGSSQEAADTPDT
M.musculus		326 TQGSKTLEEAA--EASRTSKTS-------------ESKRNCSDQAFPLGSSQED-----L
R.norvegicus	326 TPDSKTLQGTA--EAPRTSKTL-------------ESKRNCSDQALPLGSSQED-----L

S.cerevisiae	     -----------------------
N.crassa		388 TTQ-TSKPGLVAYDSDSD-----
S.pombe                  -----------------------
T.thermophila	     -----------------------
A.thaliana		     -----------------------
C.elegans		353 -----PISLIAQEYGNSSDDSD-
D.melanogaster	299 -----KPISLVGDYSSSDNDSNG
D.rerio		368 IDSCATGKSLVADYSDSDSGSEV
X.tropicalis	362 TAHTVSLSCLVPDYSDSSSESDG
H.sapiens		377 RHPCSLGSSLVADYSDSESE---
M.musculus		366 LNPNTPNASLVADYSDSESE---
R.norvegicus	366 LHPNTPNASLVADYSDSESE---

Supplementary Figure 4: Amino acid sequence alignments of CWC16a/CCDC130 proteins in some model organisms as compared to CWC16/CCDC94 in S. cerevisiae 
The amino acid sequences of CWC16a proteins belong to Family: CELL CYCLE CONTROL PROTEIN CWF16-RELATED, Subfamily: COILED-COIL DOMAIN-CONTAINING PROTEIN 130 CCDC130) (PTHR12111:SF5) in PANTHER (http://www.pantherdb.org/panther/) in a different model organisms are aligned in comparisons of CWC16 in S. cerevisiae, which belongs to Subfamily: COILED-COIL DOMAIN-CONTAINING PROTEIN 94 (CCDC94) (PTHR12111:SF4). The methods for alignment and visualization are described in the legend of Supplementary Figure 3.
