# Supplementary material for: A genetic screen implicates a CWC16/Yju2/CCDC130 protein and SMU1 in alternative splicing in Arabidopsis thaliana
Source: RNA. 2017 Jul;23(7):1068–79. doi: 10.1261/rna.060517.116 (PMC5473141; doi:10.1261/rna.060517.116)
Supplement: Supplemental Material [file supp_060517.116_Supplemental_Fig_S5_CWC16_alignments_Arabidopsis_thaliana.rtf]

Supplementary Figure 5 Kanno et al.
At1g25682    1 MSTLSAARADNFYYPPEWTPDQGSLNKFQGQHPLRERAKKIGEGILVIRFEMPYNIWCGG
At1g25988    1 -------MADNFNYPPDWTPNQSKENR---------------------------------
At2g29430    1 ------------------------------------------MAARNADLSLPMRLQCNN
At1g17130    1 ---MGERKVLNKYYPPDFDPAKLQRLR------------RPKNQQIKVRMMLPMSVRCGT
At2g32050    1 ---MGERKGLNKYYPPNFDPKQIPRIR------------KPKNQQRKIRSMVPLRIRCNT
At3g43250    1 ---MGERKNLNKYYPPDFDPKKIHRIK------------KPKNQQKKIRFMLPVRVRCNT


At1g25682   61 CSSMIAKGVRFNAEKKQV---------GNYYSTKIWSFAMKSPCCKHEIVIQTDPQNCEY
At1g25988   21 ---------------------------------------------RGNFGHKTDPQNCEY
At2g29430   19 CDNIMSKGTKFTSRVEDVIGE-------TYLGIKIFRFQIQCTNGSHEMKFRTDPKNADF
At1g17130   46 CGNYIYKGTKFNSRKEDVIGENFSFDVQTYLGIQIFRFYFKCTKCSAELTMKTDPQNSDY
At2g32050   46 CGNYMSEGTKINCREENVIGE-------TYLGIKIHRFYFKCSKCCTELILKTDPKNSSY
At3g43250   46 CGNYMSEGTKFNCRQEDVITE-------TYLGLKIHRFYIKCTKCLAELTIKTDPKNHSY


At1g25682  112 VITSGAQKKVEEYEAEDAETMELTAEQEKGKLADPFYRLEHQEVDLQKKKAAEPLLVRLQ
At1g25988   36 VITSGAQKKVEEYEAEDAETMELTAEQEKGKLADPFYRLEHQEVDLQKKKAAEPFLVRLQ
At2g29430   72 IIESGATRLLLPD-----------------------------------------------
At1g17130  106 IVESGASRNYEPWRAEDEEVDKDKQKRDAEEMGDAMKSLENRTLDSKREMDIIAALDEMK
At2g32050   99 VAESGATCVYDQHEEEEQ----------AEDGGDRMSSLEKRTLVSKREVDVMAALDEMK
At3g43250   99 TVESGASCLYNGHEDIEE--------EKKKKHENALESLENRTVVSKREIEVMASLDELK


At1g25682  172 RVSDARHA---DDYSLNKALRAQLRRHRKRVAEEETA----SRKLGLGIRLLPKSEEDIK
At1g25988   96 RVSDARHA---DGTQKTCSRRRGCFKE-ARLGEKSDFFERVKKILRLPQTLLGVIIEQEK
At2g29430      ------------------------------------------------------------
At1g17130  166 SMK-SRHATVSVDAMLEALQRTG-AEKVKRIEEEDEAV-IKSI-FGKQKEVIRRIADEEI
At2g32050  149 SMK-SRRVSVSVDSMLEDLSKRH-KEEEEVAKEEDAAL-IKSTKFGKQRRIV----DEE-
At3g43250  151 SMK-SRRASLSVDYMLEDLSRRK-KQEEENV--EEELL-IKSIKFGKRIRTD----EEK-


At1g25682  225 AASNVKFKS-KFDKNRKDKRALI----------HASSIFPESSYSSSKKRMELEAKRRKI
At1g25988  152 NGAGRKEKENNRSISIKSTEGRI----------QSFSIV---------------------
At2g29430      ------------------------------------------------------------
At1g17130  222 DDDYDDDDIDDYPSLQKEKKGSSSDLSKKRKATEVSPSNPTDILTSSSAENPKEPKKQAI
At2g32050  201 TDEMEKTKKVRM-----EA------------------------------VDEKKPKTK--
At3g43250  201 KKNYEAFDEK--------K------------------------------KKKKKPKKRDS


At1g25682  274 SAASASSLLRGGFKAS---SLSTNPSASKPK------VSSVSVRKL-----------
At1g25988      ---------------------------------------------------------
At2g29430      ---------------------------------------------------------
At1g17130  282 SKQPFKSVHIKVIKKQPQPTSSSTPAPAKPEEKKSDGAANTSLASLFQNYGSDEDED
At2g32050  224 -----KLACI--------------------ITLKKKKTTSLGLASLCHNYGADEEE-
At3g43250  223 -----GTVCI--------------------ISKKK-----TGLESLYHNCDNDSDDE


Wild-type CWC16a (At1g25682):
MSTLSAARADNFYYPPEWTPDQGSLNKFQGQHPLRERAKKIGEGILVIRFEMPYNIWCG
GCSSMIAKGVRFNAEKKQVGNYYSTKIWSFAMKSPCCKHEIVIQTDPQNCEYVITSGAQ
KKVEEYEAEDAETMELTAEQEKGKLADPFYRLEHQEVDLQKKKAAEPLLVRLQRVSDA
RHADDYSLNKALRAQLRRHRKRVAEEETASRKLGLGIRLLPKSEEDIKAASNVKFKSKF
DKNRKDKRALIHASSIFPESSYSSSKKRMELEAKRRKISAASASSLLRGGFKASSLSTNPS
ASKPKVSSVSVRKL*

cwc16a-1 mutant:
MSTLSAARADNFYYPPEWTPDQGSLNKFQGQHPLRERAKKIGEGILVIR*IIYYIISSF*L*
HREGDATFGWIHTSCCSLDCYCDP*IE**G*TLSIT*KNLFDDQM*LKCARC*SLPLVYILL
IDQKLCFCYSQFGLNFIRAFVFFRFEMPYNIWCGGCSSMIAKGVRFNAEKKQVGNYYST
KIWSFAMKSPCCKHEIVIQTDPQNCEYVITSGAQKKVEEYEAEDAETMELTAEQEKGKL
ADPFYRLEHQEVDLQKKKAAEPLLVRLQRVSDARHADDYSLNKALRAQLRRHRKRVA
EEETASRKLGLGIRLLPKSEEDIKAASNVKFKSKFDKNRKDKRALIHASSIFPESSYSSSK
KRMELEAKRRKISAASASSLLRGGFKASSLSTNPSASKPKVSSVSVRKL*

Supplementary Figure 5: Amino acid sequence alignments of CWC16 family members in Arabidopsis thaliana (top) and position of amino acid substitution in cwc16a-1 mutant (bottom)
There are six CWC16 family members in A. thaliana. At1g25682 and At1g25988 proteins belong to the Family: CELL CYCLE CONTROL PROTEIN CWF16-RELATED, Subfamily: COILED-COIL DOMAIN-CONTAINING PROTEIN 130 (CCDC130) (PTHR12111:SF5) in PANTHER (http://www.pantherdb.org/panther/) (CWC16a), while rest of the four proteins belong to Subfamily: COILED-COIL DOMAIN-CONTAINING PROTEIN 94 (CCDC94) (PTHR12111:SF4) (CWC16b). The methods for alignment and visualization are described in the legend of Supplementary Figure 3. The cwc16a-1 mutation we identified in At1g25682 (2nd intron splice site acceptor) creates a premature termination codon (red asterisk) that disrupts the ORF after approximately 50 amino acids.
