# Supplementary material for: A genetic screen implicates a CWC16/Yju2/CCDC130 protein and SMU1 in alternative splicing in Arabidopsis thaliana
Source: RNA. 2017 Jul;23(7):1068–79. doi: 10.1261/rna.060517.116 (PMC5473141; doi:10.1261/rna.060517.116)
Supplement: Supplemental Material [file supp_060517.116_Supplemental_Legends.docx]

*Supplemental Material*

Supplemental Figures

Supplemental_Fig_S1.pdf: GFP-weak mutants *gfw1* and *gfw2* are new alleles of *prp8* and *rtf2*

Supplemental_Fig_S2.pdf: Complementation of *cwc16a-1*, *smu1-4* and *smfa-1* mutations

Supplemental_Fig_S3.rtf: CWC16 alignments_plants

Supplemental_Fig_S4.rtf: CWC16 sequence alignments_model organisms

Supplemental_Fig_S5.rtf: CWC16 sequence alignments –*Arabidopsis thaliana*

Supplemental Tables

Supplemental_Table_S1.xls: CWC16 orthologs in other organisms

Supplemental_Table_S2.xls: Accumulation of GFP and NPTII transcripts not significantly altered

Supplemental_Table_S3.xls: gfp depth

Supplemental_Table_S4.xls: MES_IR

Supplemental_Table_S5.AS_ES

Supplemental_Table_S6.xls: DEGs

Supplemental_Table_S7.xls: DEGs_alternative splicing.xls

Supplemental_Table_S8.doc: Primers

Supplemental_Table_S9.xls: RNA read mapping statistics
