# Supplementary material for: A genetic screen implicates a CWC16/Yju2/CCDC130 protein and SMU1 in alternative splicing in Arabidopsis thaliana
Source: RNA. 2017 Jul;23(7):1068–79. doi: 10.1261/rna.060517.116 (PMC5473141; doi:10.1261/rna.060517.116)
Supplement: Supplemental Material [file supp_060517.116_Supplemental_Table_S8_primers.docx]

**Supplemental Table S8: Primers (5’ to 3’)** Primers used for this study are listed below.

Sequencing primers of CWC16 gene (At1g25682)

CWC16-1 TTTTGGGCTATACGAAACAC Fragment amplification

CWC16-2 AAGACATTTACCGTACCGAC Fragment amplification and sequencing

CWC16-3 ACGTGAAATTCAAGAGCAAG Sequencing

CWC16-4 GCTTGCTGATGGGTTTGTAG Sequencing

CWC16-5 CTTTCCCTTCTCTGCAAAAG Sequencing

CWC16-6 CGATCAACAAAATGTACACC Sequencing

Sequencing primers of SMU1 gene (At1g73720)

SMU1-1 GTTGAAGAACAGAGGAGTGG Fragment amplification 1

SMU1-2 CATATACTTGGTGCTTCAGG Fragment amplification 1

SMU1-3 CTGTGACTAGGATTGCTCAG Fragment amplification 2

SMU1-4 ATGGTCTCTAGGGAAAGCAG Fragment amplification 2

SMU1-5 CTGGAATGTCTGATTCAAGG Sequencing

SMU1-6 TCTTTGCAATGCGTCTCGAG Sequencing

SMU1-7 TTGATCCGCATGAGGTATGG Sequencing

SMU1-8 TGCTCAGTATGTACTCAAGG Sequencing

SMU1-9 GCATCTTCTTCTGTGGATGG Sequencing

SMU1-10 GGACCAATGAGAGGAAGAAG Sequencing

SMU1-11 CATGATGCATGATGATCCTG Sequencing

SMU1-12 TCGTGGTCACACATCTTATG Sequencing

SMU1-13 ACACTCTAACCTCCTATATC Sequencing

Sequencing primers of SmF gene (At4g30220)

SmF-1 TGGTCAAAGAGTAGAAAGCC Fragment amplification

SmF-2 GTTTGGTAATTGCTTGGTAC Fragment amplification

SmF-3 GTTCATGTAGGAGTCAACAG Sequencing

SmF-4 CATTCTTGAACAACTTGACC Sequencing

SmF-5 CTACTTGCTACCACTTGATG Sequencing

Sequencing primers of GFP gene

GFP-R1 TATCTGGGAACTACTCACAC Fragment amplification and sequencing

GFP-F1 GACAGAACTAATTATACCAG Fragment amplification and sequencing

Sequenising primers of EPRV enhancer region (GFP upstream region)

Spreading5’ AGGCTGCATCTTCAGGCATC Fragment amplification and sequencing

egfp3’ TTTACTTGTACAGCTCGTCC Fragment amplification and sequencing

gfp_out3’ CTCACCATGGATCCAGCTTC Sequencing

**RT-PCR**

Spreading5’ See above

egfp3’ See above

actin-f GCCATCCAAGCTGTTCTCTC Actin control

actin-r GGGCATCTGAATCTCTCAGC Actin control
